# Structural mechanism for tyrosine hydroxylase inhibition by dopamine and reactivation by Ser40 phosphorylation

**DOI:** 10.1038/s41467-021-27657-y

**Published:** 2022-01-10

**Authors:** María Teresa Bueno-Carrasco, Jorge Cuéllar, Marte I. Flydal, César Santiago, Trond-André Kråkenes, Rune Kleppe, José R. López-Blanco, Miguel Marcilla, Knut Teigen, Sara Alvira, Pablo Chacón, Aurora Martinez, José M. Valpuesta

**Affiliations:** 1 Centro Nacional de Biotecnología (CNB-CSIC), Madrid, Spain; 2 Department of Biomedicine, University of Bergen, Bergen, Norway; 3 Norwegian Centre for Maritime and Diving Medicine, Department of Occupational Medicine, Haukeland University Hospital, Bergen, Norway; 4 Instituto de Química Física Rocasolano (IQFR-CSIC), Madrid, Spain; 5 Present Address: School of Biochemistry, University of Bristol, Bristol, BS8 1TD UK

**Keywords:** Molecular neuroscience, Cryoelectron microscopy

## Abstract

Tyrosine hydroxylase (TH) catalyzes the rate-limiting step in the biosynthesis of dopamine (DA) and other catecholamines, and its dysfunction leads to DA deficiency and parkinsonisms. Inhibition by catecholamines and reactivation by S40 phosphorylation are key regulatory mechanisms of TH activity and conformational stability. We used Cryo-EM to determine the structures of full-length human TH without and with DA, and the structure of S40 phosphorylated TH, complemented with biophysical and biochemical characterizations and molecular dynamics simulations. TH presents a tetrameric structure with dimerized regulatory domains that are separated 15 Å from the catalytic domains. Upon DA binding, a 20-residue α-helix in the flexible N-terminal tail of the regulatory domain is fixed in the active site, blocking it, while S40-phosphorylation forces its egress. The structures reveal the molecular basis of the inhibitory and stabilizing effects of DA and its counteraction by S40-phosphorylation, key regulatory mechanisms for homeostasis of DA and TH.

## Abstract (web summary)

Tyrosine hydroxylase (TH) catalyzes the rate-limiting step in the synthesis of the catecholamine neurotransmitters and hormones dopamine (DA), adrenaline and noradrenaline. Here, the authors present the cryo-EM structures of full-length human TH in the apo form and bound with DA, as well as the structure of Ser40 phosphorylated TH, and discuss the inhibitory and stabilizing effects of DA on TH and its counteraction by Ser40-phosphorylation.

## Introduction

Tyrosine hydroxylase (TH; EC. 1.14.16.2) catalyzes the hydroxylation of L-tyrosine (L-Tyr) to L-3,4-dihydroxyphenylalanine (L-Dopa), the first and rate-limiting step in the synthesis of the catecholamines (CAs) dopamine (DA), noradrenaline and adrenaline^1^. In the brain, CAs are essential neurotransmitters and neuromodulators involved in processes such as motor control, emotion, reward, biorhythms and learning^2^. Mutations in the *TH* gene are associated with congenital TH deficiency (THD, OMIM #605407), with phenotypes ranging from L-Dopa responsive dystonia (DRD) and infantile parkinsonism to severe, complex encephalopathy with neonatal onset^3,4^. Furthermore, a deficiency in striatal TH is a hallmark of Parkinson’s disease^5^.

TH belongs to the non-heme iron- and tetrahydrobiopterin (BH4)-dependent aromatic amino acid hydroxylase (AAAH) family, which also includes phenylalanine hydroxylase (PAH) and the tryptophan hydroxylases (TPH1 and TPH2). PAH catalyzes the hydroxylation of L-Phe to L-Tyr, the first and committed step in L-Phe catabolism, and the TPHs hydroxylate L-Trp to 5-hydroxy-Trp, the rate-limiting step in the biosynthesis of the neurotransmitter serotonin. Mammalian AAAHs mainly present as homotetramers with a three-domain subunit structure (Supplementary Figs. 1a, 2a): an N-terminal regulatory domain (RD) that consists of a structured ACT (aspartate kinase-chorismate mutase-TyrA) domain preceded by a less structured N-terminal tail of varying length; a central catalytic domain (CD) that contains the active site iron and binding-sites for substrate and cofactor; and a C-terminal oligomerization domain (OD) responsible for dimerization and/or tetramerization^6,7^. Structures of rat and human PAH that include all three domains have recently been solved by X-ray crystallography^8–11^ and Cryo-EM^10^. For human and rat TH and human TPH, crystal structures encompassing CD + OD are available^12,13^. For rat TH, an NMR structure of the isolated dimeric ACT^14^ is also available (Supplementary Fig. 1b), which allowed preparation of composite models of full-length TH based on SAXS data^7,15^. These TH models present a very dynamic structure, notably at the N-terminal tail and the long loops between the RD and CD.

In humans, a single *TH* gene gives rise by alternative splicing to four very similar TH isoforms (TH1–4), with the only difference being a variable length of the flexible N-terminus^1^. In this work, we have used TH1 (named just TH from this point onward) as it is the most studied human isoform and most similar to the only TH in non-primate mammals (Supplementary Fig. 2b). Although it has the shortest N-terminus, its RD still includes ~70 residues preceding the folded ACT (residues 71–165). ACT domains are typical in many allosteric enzymes, including PAH. However, since TH is not known to be allosterically regulated, it has been suggested that this domain has lost its original function in TH while maintaining its structure throughout evolution^14,16^. The N-terminal tail (residues 1–70) is considered to be largely disordered and variable across species, however residues 40–49 are highly conserved and followed by a poly-alanine segment of variable length (residues 51–59 in humans) that seems to have appeared in evolution after the jawless fish (Supplementary Fig. 2b). This alanine-rich region has been predicted to have high helical propensity^7^.

In order to maintain CA homeostasis, the activity and conformational stability of TH are regulated at the transcriptional and translational levels, and also posttranslationally through feedback inhibition by CAs and phosphorylation at serine/threonine residues in the N-terminal tail^17,18^. TH is phosphorylated on T8, S19, S31 and S40 by several protein kinases with different site specificities^1,18^. The different phosphorylation sites regulate TH through binding to 14-3-3 proteins (S19), cellular localization to the Golgi and synaptic vesicles (S31), and activity (S40)^1,18,19^. Early preparations of TH from the adrenal medulla revealed co-purified CAs in the active site, forming a strong bidentate catecholate-Fe(III) complex^20^. TH inhibition by CAs is competitive with respect to BH4, and CAs are released from the active site either by incubation with BH4 or by phosphorylation at S40, which is performed by several kinases, including cAMP-dependent protein kinases (PKAs)^18,21,22^. Indeed, all four human TH isoforms are inhibited by DA and phosphorylated by PKA at the corresponding phosphosites to S40 in TH1 (S44 for TH2, S67 for TH3 and S71 for TH4), which decreases the affinity by DA and reactivates enzyme activity^23,24^. Thus, the strength of feedback inhibition by DA can be modulated by S40-targeting signalling pathways, e.g., enforced by inhibitory auto-receptors that lower PKA activity^25^.

DA acts not only as a feedback inhibitor, but its binding also stabilizes TH, as seen as an increased thermal stability^7,26,27^ and decreased susceptibility to proteolysis^26–28^, as well as increased lifetime of TH activity in crude extracts from rat striatum^29^. This stabilizing effect is important for maintaining TH levels in vivo, particularly in the axo-terminal compartment, as has been shown in mouse models of DA deficiency^26,30^. In addition to the direct interaction of DA with the active site iron^20^, mutagenesis and truncation studies have revealed the importance of the N-terminal tail on the high-affinity binding of DA and other CAs^31,32^. The N-terminal region is also where CA-feedback inhibition is modulated by signalling mediated S40-phosphorylation. Still, due to a lack of detailed structural information for full-length TH, a deep understanding of the molecular determinants of the interaction between TH, in particular the RD, and DA is missing. In this work, we have used state-of-the art Cryo-EM to obtain the structure of full-length human TH, both in the absence and presence of its feedback inhibitor DA, and that of S40 phosphorylated TH (THS40p), which reactivates TH. These structures provide us with a better understanding of the inhibitory and stabilizing role of DA in the regulation of TH activity and TH protein turnover.

## Results

### Three-dimensional reconstruction of the ligand-free TH (apo-TH)

**Fig. 1:**
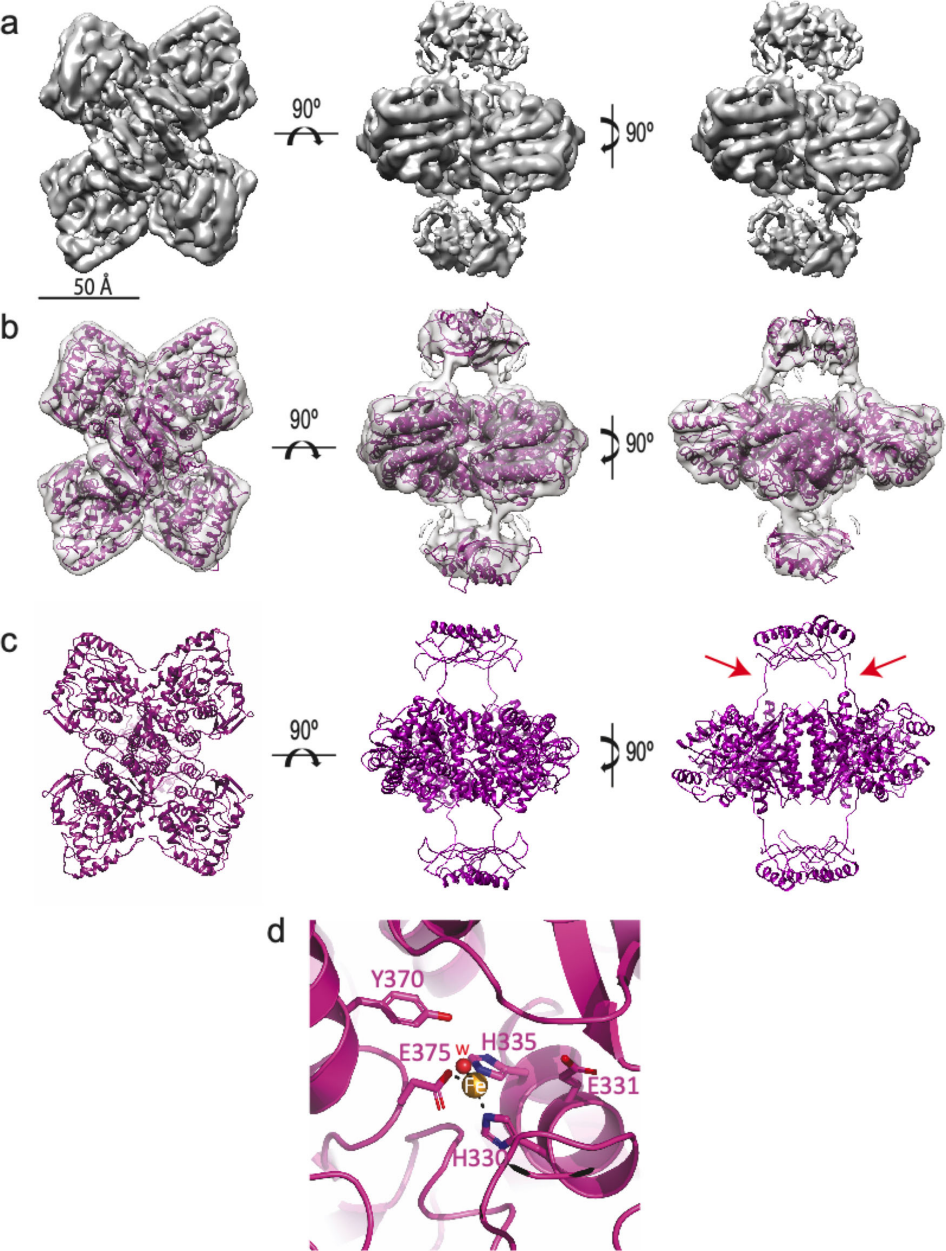
Structure of the human tyrosine hydroxylase (apo-TH). **a** Three orthogonal views of the Cryo-EM map showing the main features of the full-length apo-TH at 3.9Å resolution. The scale bar indicates 50 Å. **b** The same views with the flexible fitting with iMODfit 1.51 of the atomic model in the 3D map for further sharpening steps. **c** Atomic model of the CD + OD and RD domains built from the Cryo-EM 3D reconstruction. The connections between the RD and CD are highlighted with red arrows on one side of the structure. In the left figure, the RD has been made slightly transparent for the CD + OD domains to be better observed. **d** Detail of the active site of apo-TH showing the iron coordination with H330, H335 and E375.

The high flexibility of the N-terminal RD and of the RD-CD linker has likely been a major hurdle for structural and functional analysis of the full-length TH; to date there have only been crystal structures of truncated CD + OD domains (PDBs 1TOH and 2XSN) and the NMR structure of part of the RD domain (PDB 2MDA) (Supplementary Fig. 1b). We set out to optimize conditions for structural determination of the full-length human TH, this time using Cryo-EM^33^. Recombinant human TH was expressed and purified as in^7^ (Supplementary Fig. 3a). When analyzed by gel filtration on a Superdex 200 Increase 3.2/300, the profile of purified TH showed a main peak containing tetrameric TH preceded by a smaller octamer peak^15^ (Supplementary Fig. 3b). The tetrameric population was used for optimization using a 200 keV FEI Talos Arctica located at the Centro Nacional de Biotecnología (CNB-CSIC; Madrid), and the best grid was used for data acquisition on a 300 kV Titan Krios at the DLS-eBIC facility (Oxford) (Supplementary Fig. 3c; EPU 1.12 was used for data collection with parameters described in Supplementary Table 1). The image processing and subsequent 3D reconstruction procedures are described in detail in the Methods section and in Supplementary Figs. 4 and 5. The final map, with an estimated resolution of 3.9 Å (FSC = 0.143) (Fig. 1a), shows a central tetrameric structure formed by the CD and OD domains that is 110 Å long, 86 Å wide and 38 Å high. The four subunits of this central structure have an asymmetric arrangement that could be explained by the tetramer being formed by a dimer of dimers. The two small masses of the RDs, separated 15 Å from the main body, have a dimeric structure and a parallelepiped-like shape of 40 × 40 × 22 Å. The two masses were placed on opposite sides of the central part of the TH structure, allowing full access to the active sites of the enzyme. In the 3D reconstruction, many structural features could be assigned to α-helices and loops in the central structure formed by CD and OD domains, in particular the α-helices involved in tetramerization. In the RD domains, four α-helices were clearly visible, two of them involved in dimer formation.

However, there were areas of lower resolution, so we sought to determine the local resolution of each area of the map using MonoRes^34^ (Supplementary Fig. 4g), which showed a non-isotropic resolution distribution of the map, between 2 Å at the central part of the tetrameric structure and 10 Å at the RDs. An attempt to improve the local resolution of the latter by treating them as single particles did not result in a noticeable increase to resolution (7.1 Å), suggesting that this dimeric structure has intrinsic flexibility (Supplementary Fig. 5a). The central structure was also subjected to a further refinement by masking out the RDs, and the final resolution reached 3.4 Å (Supplementary Fig. 5b). Sharpening programs were used to improve the interpretability of the obtained map and to start the model building, as described in the Methods section and in Supplementary Fig. 5c–e. The final atomic model generated (residues 163–497) (Fig. 1c; PDB 6ZZU) was very similar to that described in the crystallographic structure of the CD + OD domains (PDBs 1TOH and 2XSN), showing the presence of iron in its active site with the coordinating residues H330, H335 and E375 (Fig. 1d)^12,35^. Surrounding residues G292, L293, A296, F299, E331, S367 and Y370 form the BH4 binding site, and R315, S323, W371, S394 and D424 form the substrate-binding site, where the latter residue is a specificity determinant for L-Tyr hydroxylation to L-Dopa in TH (Supplementary Fig. 6)^35,36^. There was also an extra density where one iron-coordinating water molecule could be placed, which was previously observed in the structure of truncated rat TH^12^. In this model, residues 160–170 corresponding to the linker between the RD and CD were also present (marked with red arrows in Fig. 1c). The final PDB file for the apo-TH tetramer included coordinates for residues 78–497 of the four subunits (PDB 7A2G), but not for the 77 N-terminal residues, which are known to be important in TH regulation.

### Structural characterization of dopamine-bound TH (TH(DA))

As TH plays a pivotal role in DA synthesis and homeostasis, it is important to understand its regulation^2,37^. The RD is essential here as it conveys communication between feedback inhibition by DA and activation by S40 phosphorylation^1^. It was, therefore, essential to characterize the RD domain structurally at the highest possible resolution and its positioning in the full-length protein. We, therefore, set out to investigate the structural differences between apo-TH and TH in the presence of DA (TH(DA)).

**Fig. 2:**
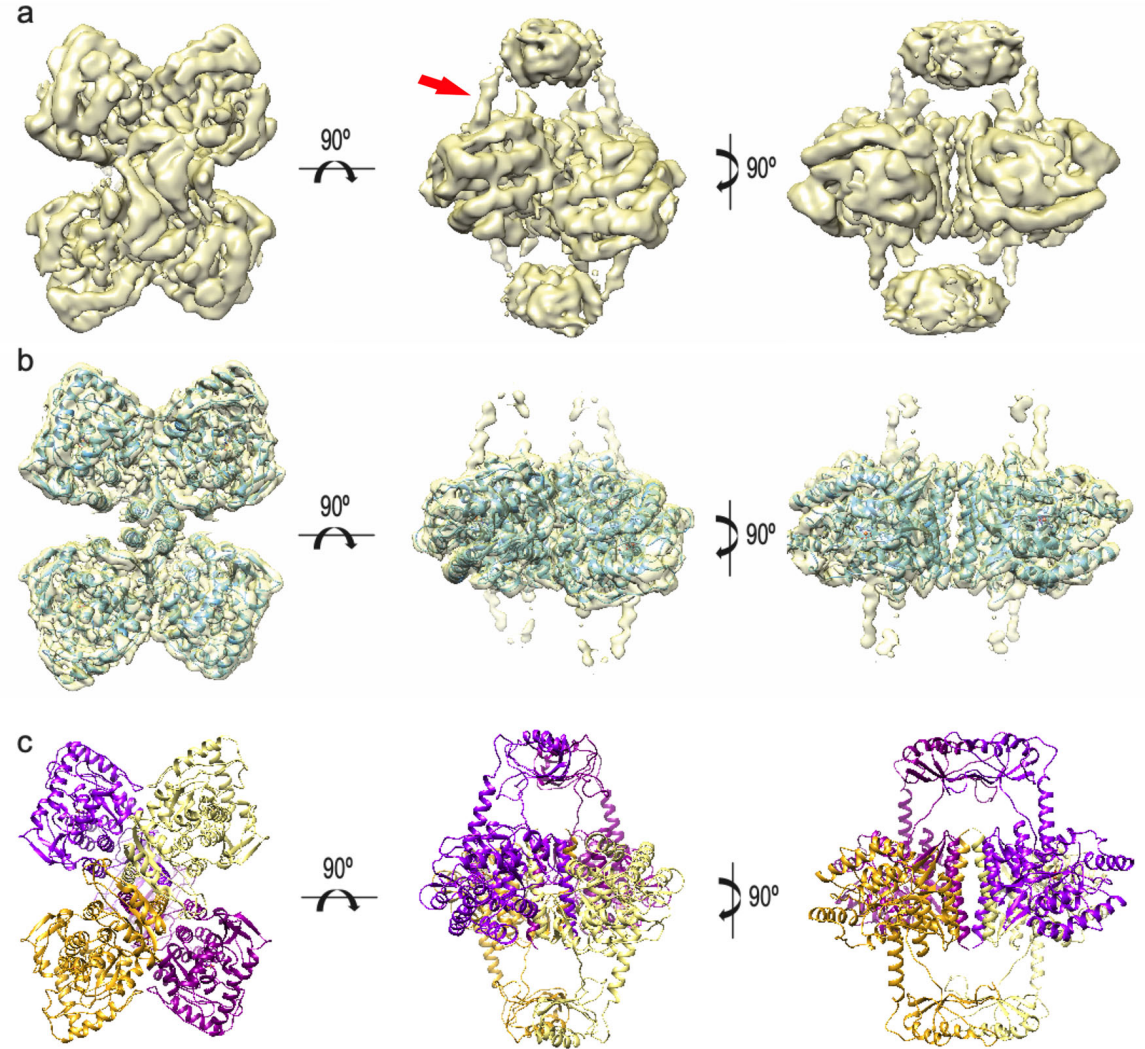
Structure of human TH in complex with dopamine (TH(DA)). **a** Three orthogonal views of the TH(DA) Cryo-EM map (4.1 Å resolution) including the RDs and masses connecting them with the CDs. The red arrow points to one of the new, helical densities connecting with the CD. **b** Atomic model of the CD + OD domains of TH(DA) docked in the 3.8 Å Cryo-EM map of the same region. **c** Predicted model of TH(DA) that includes the proposed α-helix. Each monomer is drawn in a different colour.

The formation of stable TH(DA) was carried out as described in the Methods and used for vitrification following the same conditions as for apo-TH. The cryogrids were first analyzed in our 200 keV FEI Talos Arctica, and the best one was used to record a total of 4422 movies on a 300 kV Titan Krios at the ESRF Grenoble facility (data collection parameters are described in Supplementary Table 1). The image processing and subsequent 3D reconstruction procedures followed are described in detail in the Methods section and in Supplementary Fig. 7. The final 3D reconstruction yielded a map at 4.1 Å resolution (Fig. 2a and Supplementary Fig. 7f), although analysis of the local resolution using MonoRes revealed the same differences in resolution described for apo-TH (Supplementary Fig. 7g).

The reconstruction obtained showed similar structural features and dimensions to those described for apo-TH (compare Figs. 1a and 2a), except for the presence of an extra cylindrical structure, strongly suggestive of an α-helix protruding from the central mass and contacting the RDs (red arrow in Fig. 2a). These new densities together with the connections between the RD and the CD + OD were masked, and subsequent refinement and sharpening of this part of the enzyme resulted in an increase of the resolution up to 3.8 Å (Fig. 2b).

We next carried out an atomic model building and refinement with COOT and PHENIX. Validation statistics (Supplementary Tables 2 and 3) showed reasonable correlation values for the attained resolution. We were only able to rigid-body fit a good-quality homology model (82% identity) into the lower resolution RD density. This explains the lower quality of the RD fitting in comparison with the atomic resolution modeling of the CD. For this reason, we built a model in which the side chains were omitted from the RD structure. In this map, the density of the extra cylindrical volume presented an even clearer helical shape, revealing a new α-helical region in the N-terminus (the first 70 residues) not visible in apo-TH. Unfortunately, the lower resolution of this helical region only allows to build a backbone tentative model (residues 40–497; PDB 6ZVP). Earlier secondary structure predictions and modelling by replica exchange molecular dynamics (REMD) have shown the α-helix propensity of the 45–59 alanine-rich region^7,14^. The presence of an α-helix in the 39–58 region is biochemically supported^1,31,38^ and, as described in the Methods section, the N-terminal α-helix configurations with lowest energy computed with KORP (knowledge-based orientational potential)^39^ corresponded to the 39–50 region. We nevertheless performed secondary structure predictions using PSIPRED 4.0^40^ and I-TASSER 5.1^41^, which actually pointed to a longer region of highest α-helical propensity at residues 39–58, and shorter regions in segment 13–29 with lower propensity (Supplementary Fig. 8a). Independently, we modeled a 20-residue α-helix from residues 39–58 based on the corresponding density of the Cryo-EM map (see “Methods” for details). A model for the TH(DA) tetramer comprising coordinates for residues 40–57 and 163–497 for the four subunits is also included (Fig. 2c; PDB 6ZN2) (Supplementary Tables 4 and 5).

### Structural differences between apo-TH and TH(DA)

**Fig. 3:**
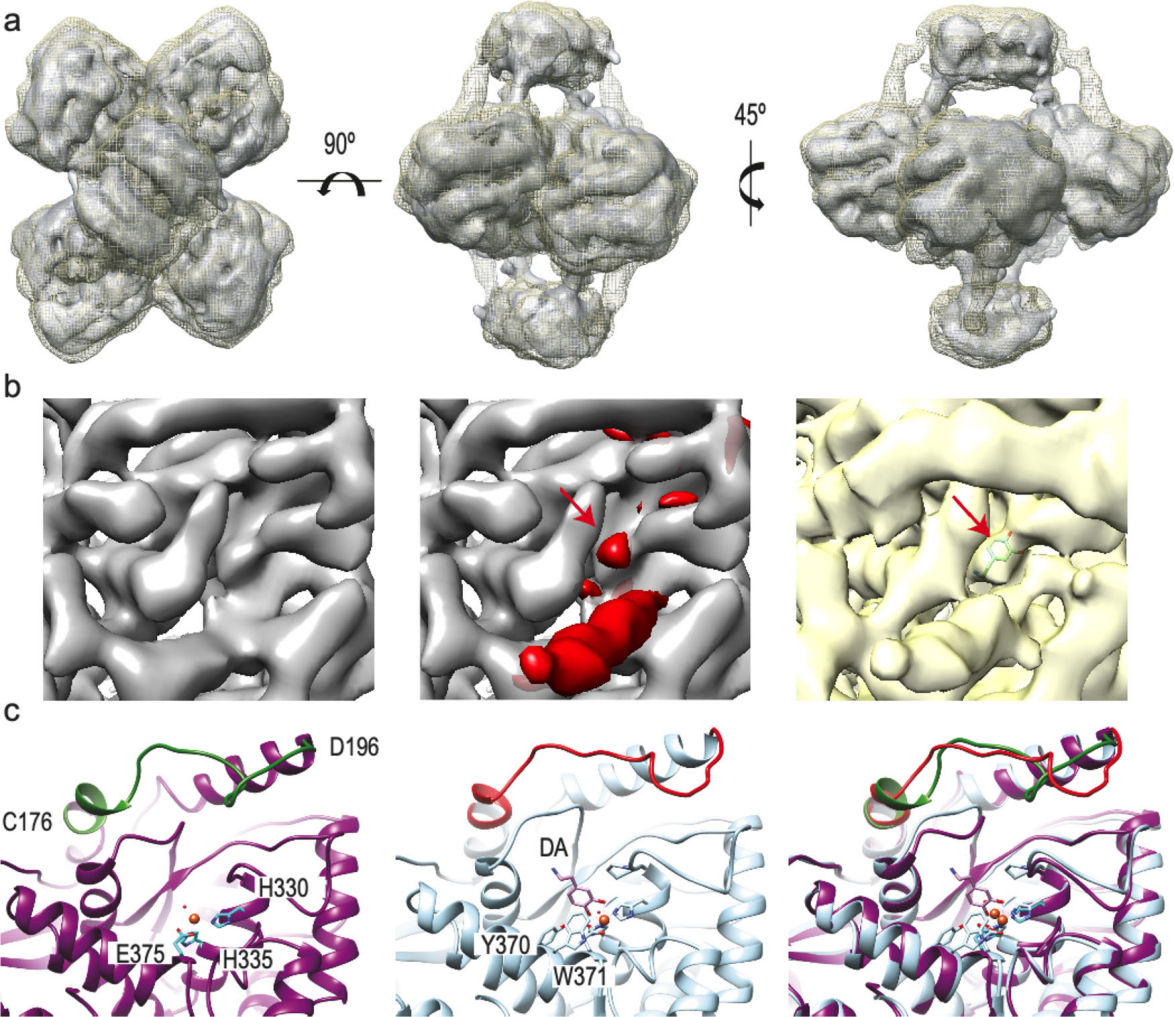
Structural comparison of apo-TH and TH(DA). **a** Three orthogonal views of the Cryo-EM maps of apo-TH (grey) and TH(DA) (yellow, mesh) showing the extra density connecting the RD with the CD active sites. **b** The same, detailed view of the active sites of (left) apo-TH, (middle) a difference map of TH(DA) minus apo-TH (red) superimposed into the apo-TH map. The red cylinder shows the internalized α-helix and the mass representing DA (red arrow). Other small masses come from changes between both conformations. The contour level is set to 3σ. The right panel shows the fitting of the atomic structure of DA into the corresponding density. **c** The same, detailed view of the atomic model of the apo-TH active site (left) highlighting the segment encompassing residues 176–196 (dark green) and those involved in iron coordination (H330, H335 and E375). In the middle, the same view of the atomic model of TH(DA) with the 176–196 segment highlighted in red. Y370, which is in the BH4 binding pocket and forms an H-bond with the bound DA, is also modelled, and the 39–58 α-helix has been removed for convenience. The right image is a superposition of the two previous images, revealing the different arrangement surrounding the active site.

Comparison of the apo-TH and TH(DA) of the CD + OD structures indicates that no large rearrangements take place upon DA binding (Fig. 3a and Supplementary Movie 1). However, there were some interesting changes, all related to the active site. When a difference map between TH(DA) and apo-TH was calculated (Fig. 3b), the major differences found were the α-helix penetrating the active site and a mass located where DA should be placed (arrow in Fig. 3b). Since the current resolution did not allow accurate fitting of DA, its atomic structure in the PAH(DA) complex (PDB 5PAH) was used to place it in the TH active site, bound to the iron with a bidentate coordination^42^. Based on how DA was placed into TH as guided by the PAH(DA) structure, DA could form hydrogen bonds with the iron-coordinating residues H330, H335, E375 and Y370 and by hydrophobic interactions with P326 (Fig. 3c). All these DA-interacting residues are conserved in PAH where they are also involved in complex formation^42^. The presence of DA was also accompanied by a rearrangement of the loop between residues C176 and D196 and a shift of the side chain of W371 towards the active-site iron (Fig. 3c). We evaluated whether the observed closure of the active site affects L-Tyr binding by alignment of the structure of substrate and BH4-bound PAH-CD (PDB 1KW0)^43^ with the TH(DA) structure (Supplementary Fig. 6a). The docked structure revealed a steric clash of DA with the cofactor, but we did not observe clashes between DA and either L-Tyr or substrate-binding residues (Supplementary Fig. 6a), in agreement with the DA inhibition being competitive only towards BH4^1,21^. Insertion of the α-helix into the active site would leave ample space for the disordered residues 1–39 to thread out of active site into the exterior (Supplementary Fig. 6b).

**Fig. 4:**
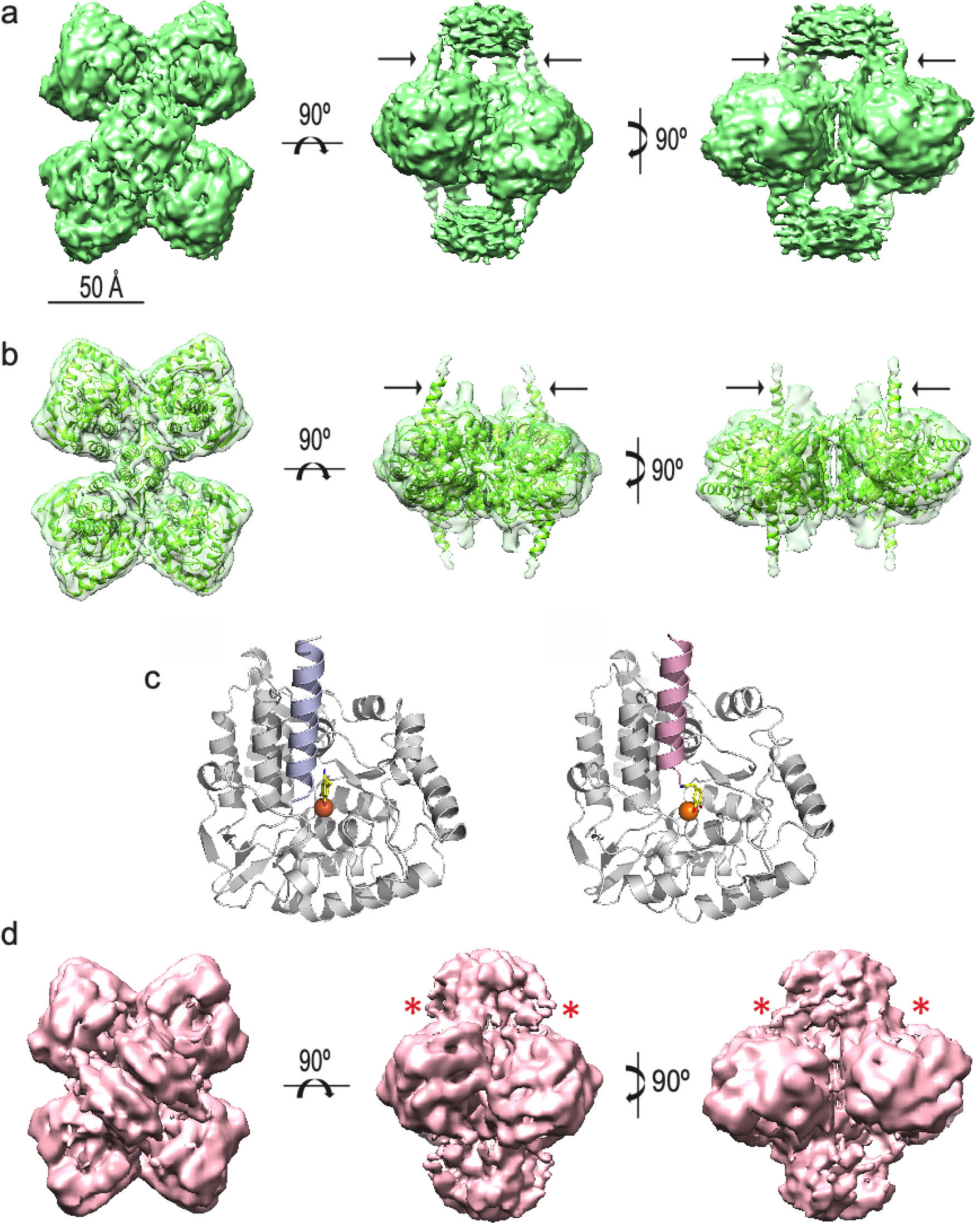
Three-dimensional reconstructions of different TH variants. **a** Three orthogonal views of the three-dimensional reconstruction of THNΔ35 in the presence of DA (THNΔ35(DA)). The black arrows point to a new mass visualized in TH(DA), which is not present in apo-TH. **b** The same views with the atomic model of CD + OD domains of THNΔ35 (the RDs have been removed) docked into the corresponding mass of the THNΔ35 three-dimensional reconstruction. The black arrows point to the α-helix entering into the active site. **c** Comparison of the atomic model of the TH active site of TH(DA) (left) and THNΔ35(DA) (right) showing the similar location of the α-helix inside the active site. **d** Three orthogonal views of the three-dimensional reconstruction of phosphorylated on S40 (THS40p). The asterisks point to the position where the N-terminal α-helix is located in both TH(DA) and THNΔ35(DA) reconstructions, but absent in this structure.

The major difference found between apo-TH and TH(DA) involves the above-mentioned presence of the N-terminal α-helix (residues 39–58) inserted into and blocking the active centre. To further confirm the presence of an α-helix in the 39–58 region of the N-terminal regulatory domain, we performed the 3D reconstruction of a mutant with the first 35 residues deleted (THNΔ35). The mutant was purified, incubated with DA (THΔN35(DA)) and vitrified as described above for apo-TH and TH(DA). A total of 13,213 movies were acquired at the Diamond Light Source (DLS) electron Bio-Imaging Centre (eBIC), and 1,626,575 particles were automatically selected and subjected to 2D and 3D classification as described for the two other 3D reconstructions. The final 3D reconstruction of THΔN35(DA)) (4.6 Å resolution) (Fig. 4a–c and Supplementary Tables 6 and 7) showed the presence, as in the case of TH(DA), of an α-helix fixed at the iron-coordinated DA in the active site, with no residues resolved upstream (arrows in Fig. 4a, b). The similarity of both 3D reconstructions (although at different levels of resolution), which reveals the presence of the α-helix in the same place as in the case of TH(DA) (Fig. 4c), reinforces the notion that the α-helix is formed by residues of the 39–58 region, and that its stabilization in the active site is associated with tight DA binding and strong TH inhibition.

### The binding of the N-terminal α-helix (residues 39–58) to DA at the active site enhances the inhibition and thermal stability of TH

**Fig. 5:**
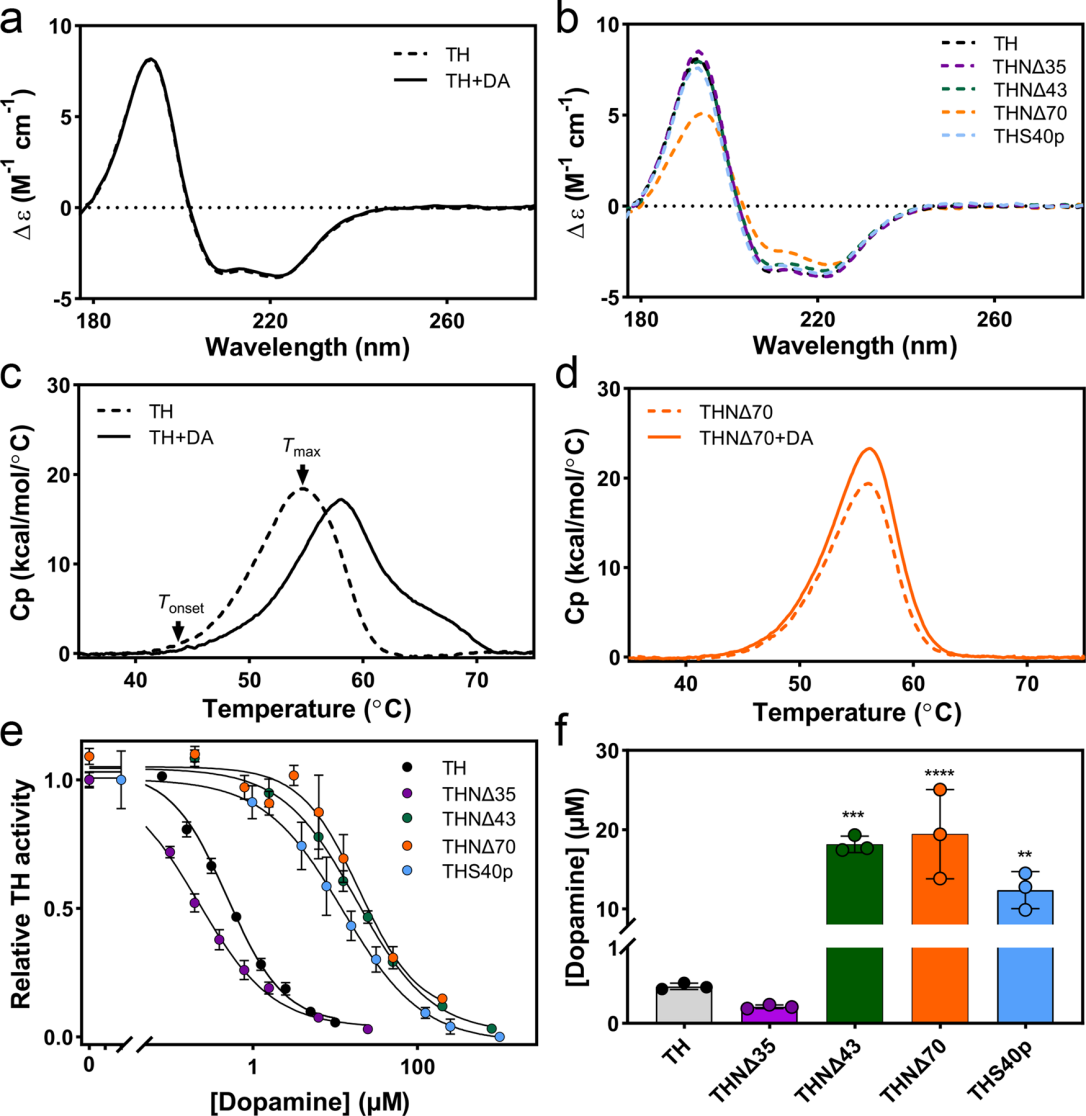
Biophysical characterization of different TH samples and inhibition of their enzyme activity by DA. Representative synchrotron circular dichroism spectroscopy (SRCD) profiles of **a** apo-TH (broken line) and TH(DA) (continuous line), and **b** apo-TH (black line), the deletion mutants THNΔ35 (pink line), THNΔ43 (green line) and THNΔ70 (orange line), and TH with phosphorylated S40 (THS40p; blue line). **c**, **d** Representative differential scanning calorimetry (DSC) profiles, in the absence (broken line) and presence (continuous line) of DA, of TH (**c**) and THNΔ70 (**d**). *T*_onset_ and *T*_max_ are indicated by arrows in (**c**). **e** Relative TH activity versus DA concentration for apo-TH (black dots), the deletion mutants THNΔ35 (pink dots), THNΔ43 (green dots), THNΔ70 (orange dots), and THS40p (blue dots). Dots represent the mean + SD (*n* = 3) and solid lines are fittings to the four-parameter logistic nonlinear regression model. **f** The bars represent the IC50 values obtained from the fitting the four-parameter logistic nonlinear regression model to triplicate curves, presented as mean ± SD, with the individual values as dots. Asterisks indicate significant changes compared to TH using one-way ANOVA followed by Tukey’s multiple comparisons test; *p* = 0.0001 (***) for TH vs. THNΔ43, *p* > 0.0001 (****) for TH vs. THNΔ70, *p* = 0.0026 (**) for TH vs. THS40p. Source data are provided as Source Data File.

The α-helix observed in the TH(DA) and THNΔ35(DA) structures could also be present in apo-TH despite not being visualised due to its great flexibility, or it could be formed upon DA binding. We, therefore, measured the secondary structure content of the two TH conformations by synchrotron circular dichroism spectroscopy (SRCD) (Fig. 5a). The far-UV spectra were completely overlapping, and calculation of the secondary structure showed no increase in helix content upon incubation with DA (Supplementary Table 8), supporting the presence of the N-terminal α-helix in apo-TH. In order to better define the helical content of the N-terminus, we generated two deletion mutants (Supplementary Fig. 8a) in addition to THΝΔ35: one lacking the N-terminal 43 residues (THΝΔ43), and another with the entire N-terminal removed (THΝΔ70). Far-UV scans (Fig. 5b) and secondary structure calculations (Supplementary Table 8) showed that whereas THNΔ35 and THΝΔ43 were almost identical to apo-TH and their secondary structure was not affected by DA binding, THΝΔ70 presented a significantly lower α-helical content. These results strengthen the notion that the N-terminal, alanine-rich α-helix is preformed and not in stable contact with any structured domain of apo-TH but gets locked into the active site groove upon DA binding, allowing its visualization by Cryo-EM.

Next, we analyzed apo-TH and TH(DA) by crosslinking mass spectrometry (XL-MS), which can provide information about nearby regions and possibly help to elucidate whether the presence of DA stabilizes the protein and produces any detectable rearrangement that would account for the presence of the α-helix. Both complexes were incubated in the presence of the crosslinker BS3 prior to trypsin digestion and LC-MS/MS analysis. The MS/MS data were searched with StavroX and the results were filtered at a false discovery rate (FDR) < 5%. We identified 300 (apo-TH) and 340 (TH(DA)) peptide-spectrum matches (PSMs) corresponding to, respectively, 128 and 120 unique crosslinked peptides. We then focused our analysis on the crosslinks detected in the flexible, N-terminal region and for that, we queried for crosslinks that mapped the first 20 N-terminal residues of TH, the most flexible region of the enzyme. The number of crosslinked peptides encompassing this region of the ligand-free complex (19 peptides, 55 PSMs) almost doubled that of the dopamine-bound one (10 peptides, 24 PSMs) (Supplementary Fig. 8b). Moreover, the crosslinked peptides observed in TH(DA) were a subset of those found in apo-TH, as no TH(DA)-specific crosslinks were identified (Supplementary Fig. 8b). Finally, two of the apo-TH-specific peptides (Supplementary Fig. 8c–e) reflected an interaction between the N-terminus of the protein and K204, a residue found in an external α-helix that surrounds the active centre. The larger number of crosslinks involving the N-terminus in apo-TH compared with TH(DA) speaks of a higher flexibility of the former, which is consistent with the presence of an immobile α-helix in the latter that would limit the movement of the entire N-terminal tail (bottom images in Supplementary Fig. 8b), indicative of increased protein stability.

We then analyzed a possible effect of DA on the thermal stability of TH and the three deletion mutants by differential scanning calorimetry (DSC) measurements, which provides the temperatures for onset of thermal denaturation (*T*_onset_) and the transition maximum (*T*_max_). The stabilization by DA, seen by delayed *T*_onset_ and increased *T*_max_, was highest for WT and *T*_*m*_ decreased with the length of the deletion, as shown in the representative scans for WT (Fig. 5c) and THΝΔ70 (Fig. 5d) and the parameters summarized in Supplementary Table 9. These results support the role of the α-helix sustaining DA-dependent stabilization.

We also performed activity assays to analyze the inhibitory effect of DA on full-length TH and the three truncated forms (Fig. 5e, f). The calculated IC50 for TH (0.49 ± 0.04 µM) agreed well with values obtained in previous studies at similar conditions^21,44,45^. The important role of a full-length α-helix in locking the active site for regulatory feedback inhibition of TH activity was revealed as a large increase in IC50 for both truncated mutants THΝΔ43 (18.1 ± 1.0 µM) and THΔΝ70 (19.4 ± 5.6 µM) while THNΔ35 shows a slightly lower IC50 (0.22 ± 0.02 µM) than TH (Fig. 5e). Altogether, our results indicate that residues at the N-terminal part of the α-helix are important for the high-affinity binding of DA.

### Modelling the structural response to S40 phosphorylation

The feedback inhibition of TH by DA is alleviated by PKA phosphorylation of TH at S40, both in vitro and in vivo^1,18,21^. We prepared S40 phosphorylated TH (THS40p), which, as expected^23,24^, presented an increased IC50 for DA (12.4 ± 2.3 µM), 25-fold higher than unphosphorylated TH (Fig. 5d, e), and not significantly different from the IC50-values measured for truncated forms THNΔ43 and THNΔ70. SRCD analysis of THS40p revealed a spectrum very similar to that of the unphosphorylated sample and comparable content of secondary structure elements (Fig. 5b and Supplementary Table 8), indicating that the decreased affinity for DA may arise from a separation of the helix from the iron-coordinated DA in the active site rather than from disruption of the helical structure. This separation is expected to result in loss of visualization of the helix in the Cryo-EM structure, as in the apo-TH structure, and to understand the structural causes for the observed effect of this phosphorylation we generated by Cryo-EM a 3D reconstruction of THS40p. Aliquots of this sample were vitrified and 9241 movies from the best cryogrid were acquired at the Diamond Light Source (DLS) electron Bio-Imaging Centre (eBIC). From these, a total of 1,610,418 particles were automatically selected and 2D and 3D classified as described in the “Methods” section. The 3D reconstruction of THS40p (Fig. 4d) shows a similar overall three-dimensional structure to that of apo-TH, TH(DA) and THNΔ35(DA) with the main point being that, as in the case of apo-TH, no α-helix is visualized blocking the active centre (asterisks in Fig. 4d).

**Fig. 6:**
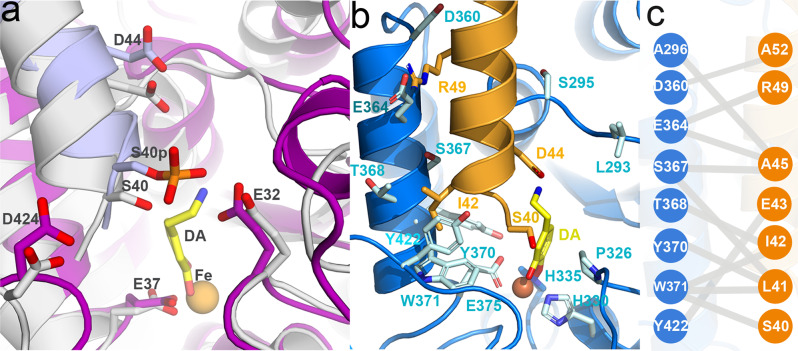
Modelling of the TH active site. **a** Modelling the effect of S40 phosphorylation on the interaction of the N-terminal α-helix with DA. Representative conformations from the last 50 ns of the 500 ns MD simulations for TH(DA) (grey ribbon) and pS40-TH(DA) (light blue ribbon). See “Methods” for preparation of the representative conformation. The resulting structures show a slight shift of the N-terminal α-helix upon phosphorylation, most probably due to electrostatic repulsion between the phosphate and E325, E375 and D424. **b** A detailed view of the atomic model of the TH(DA) active site. (left) The N-terminal α-helix (orange), which establishes connections with the adjacent helix D360-E375 and with residues of the 290–297 and 420–429 loops (blue, right). **c** A cartoon depicting the interactions established between residues of the N-terminal α-helix that enters the active site, and residues of adjacent regions.

The low affinity of THS40p for DA binding (Fig. 5e, f) hampered the preparation of homogeneous samples of DA bound to S40-phosphorylated TH (THS40p(DA)) for Cryo-EM investigation, and in order to model the initial conformation of this complex we used the 3D reconstruction of TH(DA) and modelled a phosphate group at S40 (Fig. 6a). The added phosphate did not seem a priori to clash with any other residue in the surroundings, though it was close to E325, E375 and D424, which may exert some electrostatic repulsion over the phosphate and contribute to a displacement of the helix. More insights on the possible interactions between the phosphate, DA and TH were obtained from molecular dynamics (MD) simulations on four structures (the apo forms of TH and THS40p, in addition to TH(DA) and THS40p(DA)) left it run for 0.5 µs. The four systems seemed to equilibrate within the first 200 ns and then remained relatively stable for the rest of the 0.5 µs simulations (Supplementary Fig. 9a). The ACT domain (residues 71–165) showed higher average mobility when compared to the CD, as shown by calculated backbone atom fluctuations (Supplementary Fig. 9b). After initial equilibration we monitored some selected distances, averaged for all subunits, reflecting initial changes affecting the N-terminal part of the helix and around iron and DA (Supplementary Table 10). S40-phosphorylation of TH caused slightly longer distances between the N-terminal part of the helix (D44 and S40) and both Fe and DA (notably seen in the comparison of TH(DA) with THS40p(DA)) (Supplementary Table 10). The elimination of DA (comparison of TH(DA) with TH and of THS40p(DA) with THS40p) also results in longer interatomic distances between both D44 and S40 and Fe, which together with the DA-inhibition results (Fig. 5e, f) and the Cryo-EM structure of THS40p, supports that the TH activation induced upon S40 phosphorylation is associated with the release of the α-helix that was blocking the TH active site in TH(DA).

## Discussion

The AAAHs have important functions in the synthesis of biogenic monoamines that are essential for many physiological processes, and disruption of these functions (e.g., through mutations) can lead to severe disorders^35,46^. The dynamic behaviour of the RDs, which is probably closely linked to their important regulatory roles, has until now hindered detailed structural studies. The structure of full-length TH had been elusive until now, herein solved thanks to the latest advances in Cryo-EM. The structure of human apo-TH (3.9 Å resolution) (Fig. 1) shows a planar, tetrameric core that comprises the CD and OD domains, and two smaller densities on opposite sides and 15 Å apart from the central structure. This great separation explains not only the difficulty of crystallization, but also in obtaining homogeneous preparations for Cryo-EM, which is reflected in the lower resolution obtained for the RDs (Supplementary Figs. 4g and 5a).

The high resolution reached in the TH core of the apo structure allowed building of its atomic model (Fig. 1c) and enabled comparison of this area with a previous crystallographic structure (PDB 2XSN). The comparative analysis showed differences in the region encompassing residues 176–196 (Supplementary Fig. 10a): whereas the Cryo-EM model revealed a helical and loop structure arrangement, the crystallographic structure shows only a loop that is shifted from the former. This difference could be ascribed to contacts established by this flexible loop in the crystallographic arrangement (Supplementary Fig. 10b). The presence of DA in the DA-bound TH was also accompanied by a rearrangement of the loop between residues C176 and D196 and a shift of the side chain of W371 towards the active-site iron (Fig. 3c). Nevertheless, residues 176–190 centred around F183 have indeed been shown to have an important catalytic role in TH, controlling the coupling of amino acid hydroxylation to tetrahydropterin cofactor oxidation^47^. In the equivalent Y138-centred active site loop in PAH, which is involved in enzyme activation and catalysis^10,48^, large conformational changes effected by ligand binding have also been observed.

Similar to what was found in the recent Cryo-EM PAH structure^10^, there was anisotropy in the resolution, with the RDs showing less resolution both because of their inherent flexibility and the dynamic linkers that connect them to the central structure. However, while the resolution in most of the TH core ranges from 2 to 4 Å, it was only about 5 Å in PAH. Whereas tetramers of TH (and also TPHs) form by leucine-zipper interactions, the many polar residues in the PAH OD (Supplementary Fig. 2a) may allow for different conformations and a dimer-tetramer equilibrium that would limit the possible resolution. There were also differences in the CD that have to do with its arrangement relative to the central OD, with a 12.7° outward tilt of the CD in TH compared with that of PAH (Supplementary Fig. 10c, left). By comparing the maps for TH (Figs. 1 and 2) and PAH^10^, you can clearly see: (1) RDs in TH are arranged as dimers and the ACTs are separated ~15 Å from the central part of the core structure (~30 Å from the active site); and (2) ACTs in resting-state PAH are arranged as monomers, in physical contact with the core structure (Supplementary Fig. 10d, right), and shifted 88° compared to the TH monomer (Supplementary Fig. 10c, right). In agreement with this arrangement, isolated TH RDs form a dimer^14^ (Supplementary Fig. 1b), whereas the PAH RDs are monomeric in the absence of L-Phe^49^. Allosteric substrate activation of PAH has been proposed to lead to dimerization of adjacent dimers, reaching a conformation resembling that seen in TH^8,9,11^.

The ingress and stabilization of α-helix 39–58 into the active site largely explains the tight binding of DA, since in addition to the bidentate interactions of the catechol moiety with the Fe(III) and active site residues H330, H335 and P326^12,20^, in our atomic model of TH(DA) the catecholamine establishes interactions with L41 and D44, and with Y370 in the D360-E375 α-helix (Fig. 6b). The increased thermal stabilization of TH upon DA binding as well as the low *k*_off_ for DA, which is the main rate constant affected by S40 phosphorylation^45^, are largely explained by the immobilization of the 39–58 α-helix through the interactions with DA and additional CD residues. Together with S40, DA interacting residues L41 and D44 are located at the beginning of the 39–58 α-helix, which appears as a pertinent region for TH regulation by DA. The regulatory α-helix runs parallel to and establishes contacts with the D360-E375 helix and the V290-R297 loop (A45 with A296), and with the central region of the Q420-S429 loop (I42 with Y422) (Fig. 6b). It is important to note that this model is reinforced by mutational and MS-monitored hydrogen/deuterium exchange studies that show the involvement of several of these residues in DA binding^31,38,50^. Moreover, truncation studies of the N-terminal residues have shown that absolute removal of DA inhibition requires deletion of residues ≥ 39^31^. It is however important to note that in the TH(DA) structure no residues were resolved upstream of Q39 in either TH(DA) or THNΔ35(DA). This unresolved part of the N-terminal hosts important residues for determining the conformational changes effected by phosphorylation and DA binding affinity, such as R37 and R38, which form the recognition site for PKA^1,32^. We cannot then disregard that these unobserved residues may establish interactions with TH, despite not being stabilized enough to be resolved by Cryo-EM. Furthermore, the structural fixation of the N-terminal α-helix could explain the reported inhibitory effect of DA on S40 phosphorylation rate^21^.

The structural stabilization of the N-terminal residues 39–77 in the DA-bound state, both in full-length TH and THNΔ35, was necessary for their visualization, as they were not observed in the apo-TH state (whose structural model starts at the ACT domain) or in the THS40p conformation. Moreover, the detailed structural information on the N-terminus provided by the TH(DA) and THNΔ35(DA) Cryo-EM structures permitted us to perform MD simulations to obtain additional structural insights on the feedback inhibition by DA and its release by S40 phosphorylation. Physiologically, TH is activated when S31 or S40 are phosphorylated, but only S40 phosphorylation affects CA binding, leaving this site as the main target for signal-mediated activation of TH^18^. In our structural model of TH(DA), S40 was placed on the N-terminal side of the α-helix, at the base of the active site. The MD-simulated phosphorylated structures pointed to an electrostatic repulsion from the nearby E325, E375 and D424 as a possible mechanism for the separation of the helix (Fig. 6a). This offers a structural explanation to the functional result since the release of the α-helix and a lower affinity for DA would result in higher TH activity (Fig. 5e).

**Fig. 7:**
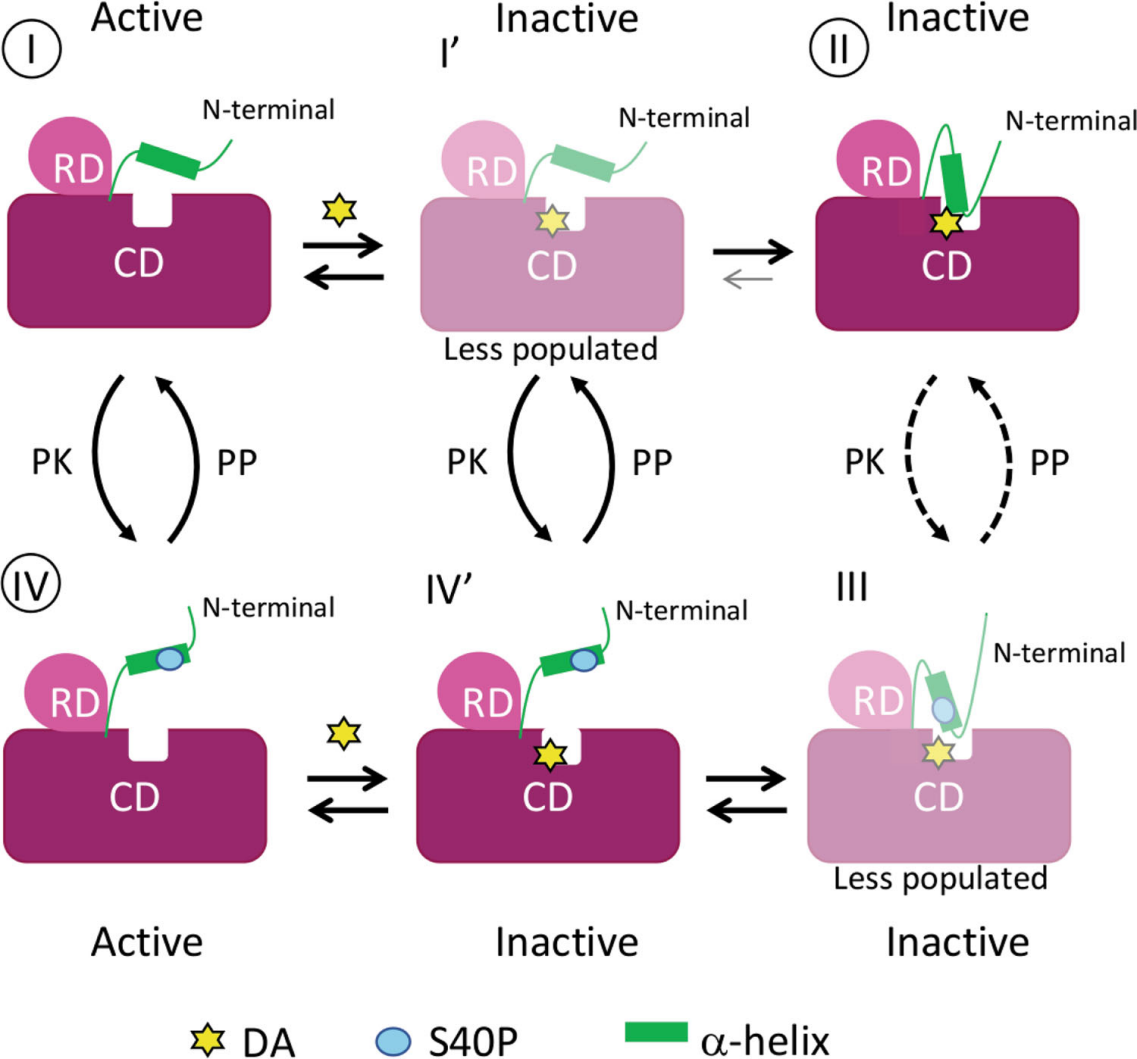
Cartoon model of DA-mediated feedback inhibition of TH and its regulation by S40 phosphorylation. In the active, apo and non-phospho state, the 39−58 α-helix of TH is detached from the main structure (I, apo-TH). The feedback inhibitor DA binds to the TH active site, most likely in the open conformation (I′, TH(DA)). DA-binding favours interaction of the N-terminal α-helix with the same binding site, which blocks DA exit and contributes to the high-affinity binding and strong inhibition of TH activity (II, TH(DA)). Protein Kinase (PK) phosphorylation of S40 in TH(DA), leads to state III (THS40p(DA)), prompting the detachment of the α-helix from the TH active site (IV′), which opens up for DA-dissociation and activation (IV, THS40p). PKs and protein phosphatase(s) (PP) control the transition between THS40p and unphosphorylated TH for both DA bound (I′ ↔ IV′ and II ↔ III) and apo-TH (I ↔ IV). States I′ and III are expected to be only transiently populated during DA binding as state II and IV' will be more stable^21,51^ (see main text). To indicate that our data do not support the presence of stable or well populated states I′ and III, these are faded in the figure. S40 is also expected to be less accessible in state II than in state I (see main text), which is indicated by stippled lines for phosphorylation of TH in state II. The case for dephosphorylation is not known, but it could be expected that state III is a poorer substrate for PP than the open states IV′ and IV. The dephosphorylation reaction III → II is therefore also stippled. The states where we provide structural details in this work (I, II and IV) are marked with circles.

All these results allowed us to propose a model in which TH is in an active conformation when the N-terminal region (which includes the α-helix) is free and detached from the main structure (Fig. 7, I). DA coordination to the TH iron (Fig. 7, I → I′) favours the interaction of the α-helix with DA and the active site (Fig. 7, I′ → II and Fig. 6b). Phosphorylation of S40 (Fig. 7, II → III) would initiate, through electrostatic and steric repulsion, the egress of the helix from the active centre (Fig. 6a; Fig. 7, III → IV′). Subsequent release of DA would result in an active TH (Fig. 7, IV′ → IV). Thus, based on data presented here and, in the literature, we expect that state I′ is only transiently populated during DA binding as state II will be more stable. Similarly, for THS40p(DA) we expect that state III is destabilized and less populated than state IV′. This model is supported by kinetic studies where S40 phosphorylation mainly increases the dissociation rate constant (*k*_off_) of DA^51^ and the mobility around the phosphorylation site^45,52^. DA binding to TH inhibits PKA-mediated S40 phosphorylation, mainly by increasing the *K*_M_ for TH, suggesting that S40 is less available in state II than in state I^21^. However, as the inhibition of TH phosphorylation by DA is not as strong as the inhibition of TH activity, we cannot exclude that S40 can be accessed in the closed conformation (Fig. 7, II)^21,51^.

Our results also highlight the importance of the α-helix in the regulation of neuronal DA homeostasis for unphosphorylated TH, as an IC50 ≈ 0.5 µM fits well with IC50-values obtained in physiologically relevant systems, such as rat striatal synaptosomes^53^. Cytoplasmic DA concentrations sufficient for a functional feedback inhibitory effect (low µM) are in agreement with the free cytoplasmic concentration of DA in neurons (~2 µM)^54^. As is the case for the deletion of α-helix 39–58(THNΔ70) or only its N-terminal part (THNΔ43), S40 phosphorylation leads to a great increase (approx. 25-fold) of the IC50 value for DA inhibition, in agreement with values obtained in previous studies^21^ (Fig. 5e, f). Thus, the DA-mediated TH inhibition was not totally eliminated upon S40 phosphorylation, but rather seems to adjust the set point of cytosolic DA to a higher level depending on the signalling strength. Previous studies have shown similar mechanisms for inhibition by different CAs and their release by Ser40 phosphorylation^21,45^, with a small difference in affinity among DA, epinephrine and norepinephrine, which might be explained by the similar location of the catechol group and the primary amine, but a slight difference in charge distribution at this amine.

DA signalling in the brain occurs mainly through volume transmission, meaning that diffusion vs. reuptake kinetics control the spread of released DA^55^. Reuptake by the DA transporter also provides neurotransmitter that can be stored in vesicular pools and reused. In the absence of feedback inhibition, the DA synthesis flux would become unaffected by the accumulating cytosolic DA at full vesicular pools, and the steady-state level of cytosolic DA would be determined by the balance between the unconstrained synthesis rate and DA metabolization by high-*K*_M_ monoamine oxidase. However, DA and other CAs are redox-active and considered neurotoxic at increased levels^56^, which may be a reason for the evolution of a strong feedback inhibition of TH, not found for serotonin and TPH. The presence of a flexible N-terminal region in TH is important for the stabilization of DA feedback inhibition and allows for an additional level of regulation, as the S40 site enables signalling pathways to modulate the feedback inhibitory strength of CAs. The CD-interacting α-helix 39–58, containing the S40 site, seems to be a key structural determinant for this regulatory crosstalk and it is unique to TH among the AAAHs (Supplementary Fig. 2a). Despite the low TH sequence identity among different organisms, the helix is likely to be highly conserved from fishes to primates with only small variations in the poly-alanine region^14^, but not in non-vertebrates where the feedback regulation by DA is different or absent^57,58^ (Supplementary Fig. 2b). The S40 PKA site is however older, but has an unresolved function in *C. elegans*^57^, whereas in *D. melanogaster* PKA phosphorylation activates the brain TH isoform, but the epidermal isoform is only activated in the presence of DA^58^. The fully functional DA ↔ S40p structural motif seems to have had a step-wise evolution; first establishing an activating S40-phosphosite, then co-evolving higher DA binding affinity that may rely on a stable helical structure to minimize entropy loss. In vertebrates, as a result of the structural N-terminal regulatory cross-talk motif, phosphorylation-mediated modulation of feedback inhibition would allow rapid anticipatory adjustment of TH activity to changes in neuronal activity, either through stimulatory or inhibitory receptors. Thus, dopaminergic autoreceptors inhibit cellular PKA activity and increase the strength of DA feedback according to levels of extracellular transmitters^25^, allowing TH activity to adapt to the anticipated lower need of DA as a result of high extracellular DA.

THD is an autosomal recessive parkinsonian disorder caused by mutations in the *TH* gene, which are registered in the PND database (www.biopku.org/pnddb/). The protein numbering in the PND database is for the longest human isoform, TH4, which is 31-residue longer than TH1. The effects of the mutations on TH activity, stability and oligomerization have been previously studied for a large number of mutants^3,15^. Analyses based on prokaryotic expression and characterization of mutation-associated effect on thermal stability, protein solubility and residual TH activity have provided a good correlation to phenotype severity for a large number of type A (L-Dopa-responsive dystonia) mutations, but less so for some of the severe type B (non-responsive infantile parkinsonism) mutations^3,4^. As seen in Supplementary Fig. 11a, where the mutations are mapped on the TH(DA) subunit structure and coloured according to their presence in type A or B patients^3,4^, the mutations are spread over the CD and OD, and the more severe type B appear located closer to the active site cavity. As DA-mediated TH stabilization largely determines the steady-state levels of TH and DA in vivo^26,30^, the full-length TH(DA) structure obtained in this work improves our understanding of the structure-based pathogenic mechanism in THD. In particular, the interactions of helix 360–375 with the N-terminal helix 39–58 and loop 290–297 around the active site (Fig. 6b and Supplementary Fig. 11b) appear relevant to understand the deleterious type B THD mutations R297W and T368M (R328W and T399M in TH4). R297 is at the centre of a crucial H-bonding and electrostatic network, and T368 interacts with I42 in the N-terminal helix (Fig. 6b and Supplementary Fig. 11b). Mutations in these residues are expected to affect the proper localization of the N-terminal helix upon DA binding, further affecting TH stabilization.

This work constitutes a significant step forward in the knowledge of the structure, regulation and stabilization of TH through feedback inhibition by DA and its reversal by PKA-mediated S40 phosphorylation. Determining the full-length structure of apo-TH also allowed us to corroborate the differences in oligomeric organization of the RDs in resting-state AAAHs. The dimeric assembly of the RDs in TH is consistent with the stable tetrameric structure of the protein, and for setting the stage for the far N-terminal tail (residues 1–70) as a main player in TH regulation. A major path in future studies remains the region preceding the 39–58 α-helix. The first 40 residues have not been visualised in this work and yet some of them (e.g., T8, S19 and S31) are important players in TH regulation.

In any case, the new structural knowledge allows for a more detailed mechanistic understanding of key physiological regulation of TH and contributes to improve the genotype-phenotype correlations in THD. A better structural understanding may also pave the way to the development of novel stabilizing/chaperoning therapies to address the deleterious neurological manifestations, very much needed by patients with L-Dopa-unresponsive THD type B. Pharmacological chaperones, which are target-specific small molecules that stabilize functional states and/or facilitate folding of non-native intermediates, have shown therapeutic potential by recovering function in mutant proteins, including phenylketonuria-associated PAH mutants, and are often discovered through high-throughput target-based experimental and/or virtual screenings^59,60^. Virtual screening and structure-based drug design are preferably carried out with 3D-structures with a resolution ≤ 3 Å for the target protein^61^. In the case of cryo-EM structures with near-atomic resolution (3–5 Å), as is the case for our TH structures, a combination of MD simulations and ensemble docking protocols appears to provide a promising strategy for their successful application in structure-based drug discovery^62^.

## Methods

### Expression and purification of the TH variants

TH with an N-terminal His-MBP tag containing a TEV protease-site was expressed from the pETMBP1a/TH plasmid^7^, and purified with amylose resin affinity chromatography. The HisMBP-TH fusion protein was eluted with 10 mM maltose, followed by cutting with TEV protease (1:50 w/w for 1 h at 4 °C). Subsequent isolation of the tetrameric TH by size-exclusion chromatography using a HiLoad™ Superdex™ 200 prep grade column (1.6 cm × 60 cm) in 20 mM Na-Hepes pH 7, 200 mM NaCl. Constructs for THNΔ35, THNΔ43 and THNΔ70 were derived from the pETMBP1a/TH construct (Genscript) and also expressed and purified as tetramers essentially as TH^7^. For synchrotron circular dichroism (SRCD), the SEC was performed in 50 mM Na-Phosphate pH 7. Protein concentrations were determined using a Nanodrop UV–Vis spectrophotometer and the theoretical extinction coefficients.

### Preparation of stable TH(DA) complex

Once TH was purified and its functionality assessed by determining the enzyme activity at standard conditions and 37 °C (see section ‘*Assay of TH activity and inhibition by DA*’, below), providing a specific activity of 1630 ± 184 nmol L-Dopa/min/mg (mean ± SD, *n* = 5 independent samples), the protein was incubated with iron and DA to obtain the stable Fe(III)-catecholate complex^20,63^. Essentially, ferrous ammonium sulfate (FAS) was dissolved in degassed water, added to TH (at a molar ratio 1:1 FAS:TH subunit) and incubated for 2 min at room temperature (RT) before DA addition (2:1 DA:TH subunit) with subsequent incubation for 3 min.

### Phoshorylation of TH on S40

TH was phosphorylated on S40 (control without kinase)^3^, using the catalytic subunit of cAMP-dependent protein kinase (CatPKA, kind gift from S.O. Døskeland^64^). TH (4 mg/ml) was assayed in 50 mM Na-β-glycerophosphate pH 7.5, 1 mM DTT, 0.1 mM EGTA, 1x protease inhibitor cocktail (without EDTA, Pierce), 0.5 mM ATP, 5 mM MgCl_2_ and incubated with the catalytic subunit of CatPKA (200 nM, diluted from 20 µM in 60% glycerol, 0.5 mg/ml soya bean trypsin inhibitor) for 30 min at 25 °C. Phosphorylation stoichiometry (0.98 ± 0.03; mean ± SD, *n* = 3 independent samples) was measured by radioactive [^32^P]-γ-ATP^3^. After phosphorylation, H89 (Sigma) (5 µM) was added and the samples were exchanged into 20 mM Hepes pH 7.0, 200 mM NaCl.

### Synchrotron circular dichroism (SRCD) spectroscopy

Small aliquots of concentrated TH (apo-TH, THNΔ35, THNΔ43, THNΔ70, and THS40p) were diluted to 0.5 mg/ml in 50 mM Na-phosphate pH 7 with a two-fold molar ratio of iron and with two-fold molar ratio of DA when appropriate. SRCD spectra were collected as from 280 to 170 nm (3 scans averaged) at 25 °C on the AU-UV beamline at ASTRID2, part of the Institute for Storage Rings (ISA) facility at the University of Aarhus, Denmark. All spectra for baselines and samples were measured in the same cuvette, a 121-type closed cylindrical cell with 100 µm pathlength. The path length of the cell was confirmed using an interferometry method and found to be 100.1 µm. The protein concentration was measured using Abs_280nm_ prior to the measurements and more accurately by *A*_205nm_ from the absorption spectra measured simultaneously with the CD spectra, using the respective theoretical extinction coefficients^65^. The *A*_205nm_ measurements were used in the conversion of raw CD units to ∆ε(M^−1^cm^−1^) values. Secondary structure content was determined in the 250–180 nm range using the BeStSel algorithm^66^.

### Differential scanning calorimetry (DSC)

A PEAQ-DSC automated differential scanning calorimeter (Malvern Panalytical) was used to obtain the melting profile of the TH proteins with and without DA. In all experiments, 9 μM TH in 20 mM Na-Hepes pH 7, 200 mM NaCl was used, with the same buffer as reference, heating from 25 to 75 °C at a scan rate of 200 °C/h. A two-fold molar ratio of iron was added to all the samples and a two-fold molar ratio of DA or water when appropriate. DA was added after 2 min incubation with FAS at room temperature. The DSC thermograms were buffer-subtracted and normalized on scan rate and concentration and analyzed in the PEAQ-DSC 1.61 analysis software. *T*_max_ was taken as the temperature with maximum Cp-value and *T*_onset_ at 5% of *T*_max_ pre-transition.

### Assay of TH activity and inhibition by DA

The activity of purified TH and truncated forms was assayed at 37 °C^7^. The purified enzymes were kept on ice and diluted with 0.5% (w/v) bovine serum albumin in 20 mM Na-Hepes pH 7, 200 mM NaCl and centrifuged at 10,000 × *g* for 5 min at 4 °C. The standard assay mixture contained 20 mM Na-Hepes, pH 7, 0.1 mg/ml catalase, 10 µM FAS, 50 µM L-Tyr and 0–800 µM DA. TH was added to a final concentration of 1 µg/ml (17 nM subunit) and preincubated at 37 °C for 1 min. The reaction was started by adding 200 µM BH4 and 5 mM DTT and stopped by adding one volume of 1% (v/v) acetic acid in ethanol after 5 min for TH, THNΔ35, THNΔ43, and THS40p and after 2 min for THNΔ70. Protein was removed by precipitation at −20 °C for 90 min followed by centrifugation at 20,000 × *g* for 14 min at 4 °C. The amount of L-Dopa in the supernatant was determined using a 1200 series high performance liquid chromatography (HPLC) system (Agilent technologies). The chromatographic separation was obtained using an Agilent Zorbax 300-SCX column with 20 mM HAc pH 3.5, 2% (v/v) propanol as mobile phase at a flow rate of 3 ml/min. The fluorescence detector was set to λ_ex_ = 280 nm and λ_em_ = 314 nm.

### Crosslinking experiments and mass spectrometry analysis

Apo-TH and TH(DA) were subjected to chemical crosslinking coupled with mass spectrometry (XL-MS). Approximately 10 μg of apo-TH and TH(DA) were incubated with 3 mM of the crosslinker Bis(sulfosuccinimidyl suberate) (BS3) in 50 mM Na-Hepes pH 7, 200 mM NaCl for 40 min at RT. The reaction was quenched for 15 min at RT by adding 50 mM Tris-HCl pH 7.0. Purified BS3-crosslinked proteins were incubated in Laemmli sample buffer (0.02% [w/v] bromophenol blue, 2% [w/v] SDS, 10% [v/v] glycerol, 60 mM Tris-HCl pH 6.8) for 5 min at 96 °C and loaded onto a 12% polyacrylamide gel. Electrophoresis was stopped as soon as the protein sample reached the top region of the resolving gel. The gel was then stained with Quick Coomassie (Generon) and the coomassie-stained band was excised and subjected to automated reduction, alkylation with iodoacetamide and trypsin digestion in a Proteineer DP robot (Bruker Daltonics). The resulting peptide mixture was speed-vac dried and re-dissolved in 0.1% (v/v) formic acid.

The LC-MS/MS analysis was carried out using a nano-LC Ultra HPLC (Eksigent, Framingham, MA) coupled online to a 5600 triple TOF mass spectrometer (AB Sciex, Framingham, MA) through a nanospray III ion source (AB Sciex) equipped with a fused silica PicoTip emitter (10 µm × 12 cm; New Objective, Woburn, MA). Chromatography was performed at a flow-rate of 250 nl/min at 50 °C under the following gradient elution conditions: 2% B for 1 min, a linear increase to 30% B in 109 min, a linear increase to 40% B in 10 min, a linear increase to 90% B in 5 min and 90% B for 5 min. The ion source was operated in positive ionization mode at 150 °C with a potential difference of 2300 V. Each DDA cycle included a survey scan (350–1250 m/z) of 250 ms in high resolution mode and a maximum of 25 MS2 spectra (100–1500 m/z) in high sensitivity mode.

For peptide identification, raw MS data were searched against a custom-made database containing the sequences of the proteins in each sample. The MS/MS ion search was performed with Stavrox 3.6.6^67^. Search parameters were set as follows: trypsin as enzyme allowing 2 and 3 missed cleavages for R and K, respectively, BS3 as crosslinker, MS tolerance of 20 ppm and MS/MS tolerance of 40 ppm, carbamidomethylation of cysteine as fixed modification and oxidation of M as variable modification. Peptide identifications were filtered at a FDR < 5%.

### Sample preparation for Cryo-EM

Purified apo-TH was subjected to a second size exclusion chromatography step to ensure protein homogeneity and stability. The sample was loaded onto a Superdex 200 Increase 3.2/300 (GE Healthcare) equilibrated with the same buffer at 4 **°**C. Fractions of 30 μl were collected in an ÄKTAmicro (GE Healthcare) device. Cryo-EM grids were immediately vitrified using a Vitrobot Mark IV (FEI). Quantifoil UltraAufoil 1.2/1.3 300 mesh grids were previously glow-discharged for 30 s at 15 mA. Aliquots of 3 μl of the different samples were incubated onto the grids, blotted for 1 s at 4 **°**C and 95% humidity and plunged into liquid ethane. TH(DA), THNΔ35(DA) and THS40p were directly vitrified under the same conditions with no extra size exclusion chromatography step.

### Cryo-EM data acquisition

All the samples were first checked using a 200 kV FEI Talos Arctica equipped with a Falcon III direct electron detector at the Centro Nacional de Biotecnología (CNB) cryo-EM facility. Data acquisition for apo-TH was performed at the Diamond Light Source (DLS) electron Bio-Imaging Centre (eBIC) using a Titan Krios electron microscope operated at 300 kV, equipped with a Gatan Quantum K2 Summit direct electron detector operated in counting mode. A total of 3867 movies were acquired at a nominal magnification of 130,000× (corresponding to a pixel size of 1.047 Å/pixel), with a defocus range of −1.6 to −2.2 μm. Movies were fractionated to 36 frames with a total exposure of 9 s. The electron dose was 39.6 e^−^/Å^2^ for a total dose of 1.1 e^−^/Å^2^ on the specimen (Supplementary Table 1).

Movies of TH(DA) were collected on a FEI Titan Krios electron microscope (Krios 1) operated at 300 kV, equipped with a Gatan Quantum K2 Summit direct electron detector mounted on a Gatan Bioquatum LS/967 energy filter at the European Synchrotron Radiation Facility (ESRF) in Grenoble. Data collection was carried out with a 130,000× nominal magnification (yielding a pixel size of 1.053 Å/pixel), with a defocus range of −1.8 to −3.2 μm. A total of 4422 movies were recorded and each movie was fractionated to 40 frames with a total exposure of 6 s. The electron dose was 37 e^−^/Å^2^ on the specimen (Supplementary Table 1).

Data acquisitions for THNΔ35(DA) and THS40p were performed at the Diamond Light Source (DLS) electron Bio-Imaging Centre (eBIC) using a Titan Krios electron microscope operated at 300 kV, equipped with a Gatan Quantum K3 detector operated in counting mode with 0.5X-binning (super-resolution mode). A total of 9241 and 13213 movies were acquired for THΔ35(DA) and THS40p respectively, at a nominal magnification of 81,000× with a defocus range of −1.6 to −3.4 μm. Movies were fractionated to 40 frames with a total exposure of 2 s for both samples. The electron dose was 30 e^−^/Å^2^ (Supplementary Table 6). Motion-corrected movies were extracted and binned 2× to the physical pixel size of 1.07 Å/pixel.

### Image processing and three-dimensional reconstruction

Image processing of apo-TH, TH(DA), THΔ35(DA) and THS40p samples was performed following a similar workflow. All programs used to obtain the different 3D models are implemented in the Scipion 2.0 software platform^68^. First, the beam-induced motion of the movies was corrected using the MotionCorr2 software^69^. After movies alignment, the contrast transfer function (CTF) was calculated and corrected by Gctf (Supplementary Figs. 4a and 7a)^9^. Particles were automatically picked with Xmipp3 –auto-picking software^70^. To save computational time during the first processing steps and to increase the signal to noise ratio, particles were downsampled by a factor of 4, and extracted with Xmipp3 – extract particles^70^. The extracted particles were subjected to a first 2D classification using Relion 2.0 (Supplementary Figs. 4b and 7b)^71^ and the best classes were subjected to several further rounds of 2D classification, allowing a much better detection of bad particles such as aggregates or particles that were very close to each other.

For apo-TH and TH(DA), different de novo initial models (Supplementary Figs. 4c and 7c) were obtained using both EMAN2^72^ and RANSAC^73^. Another initial model was obtained by low resolution (60 Å) filtering of the atomic structure of the CD of the human TH (PDB 2XSN). In the case of apo-TH, the first rounds of 3D classification were performed using Relion 2.0 without any symmetry imposition and using the different initial models (Supplementary Fig. 4d). No significant differences were found among the best class obtained from the low-pass filtered atomic structure and the de novo initial model. Particles belonging to that class were subjected to refinement using Relion 2.0— 3D auto-refine software. Since clear symmetric features were observed in this class, we sought to determine whether C2 or D2 symmetry was applicable and could contribute to better define our 3D models. The application of C2 symmetry resulted in different classes showing good arrangement in the CD and OD, but the mass corresponding to the RD was distorted showing artefactual densities. On the other hand, the D2 symmetry maintained the RD size and shape as expected according to its atomic structure (PDB 2MDA)^14^ and to the best volume obtained before symmetry imposition (Supplementary Fig. 4d). In the case of TH(DA), D2 symmetry was directly applied for 3D classification (Supplementary Fig. 7d). The particles selected after refinement (250,712 particles for apo-TH and 125,033 for TH(DA)) were re-extracted at the original pixel size to continue the image processing. Auto-refine with the original particles (Supplementary Figs. 4e and 7e) rendered a final 3D map at 3.9 and 4.1 Å resolution for apo-TH and TH(DA), respectively, as estimated by the Fourier shell correlation (FSC) method, with a cut-off of 0.143 (Supplementary Figs. 4f and 7f)^71^. This approach calculates the cross-correlation between two halves of the 3D map in the spatial frequency shells to give the resolution, but it does not contemplate the flexibility of the protein. For each 3D map, local resolution was then calculated using Xmipp3—MonoRes^34^ (Supplementary Figs. 4g and 7g). The same sets of particles were also subjected to Relion Bayesian particle polishing, however, no improvement in resolution was observed. To improve the low resolution found in the RD, these domains were extracted and processed as single particles to generate a localized reconstruction of the RD^74^ (Supplementary Fig. 5a). The final refined map at 3.9 Å resolution was used for subtracting the region of interest. First, a mask surrounding the selected part of the map (subparticles from now on) was generated and used to extract the subparticles. An initial volume was generated with Relion-reconstruct^71^ and used for 3D-classification of the subparticles. The best class was auto-refined to improve the quality of the data and the resolution. The RD final map resolution obtained reached 7.1 Å. This approach was not successful for TH(DA), probably due to the extra density affecting the proper alignment of the subparticles. Another way of improving the different domains in a protein complex is by masking the density of interest. This approach was used to increase the resolution in the CD and OD. Starting from the best auto-refined volume, a mask was generated that excluded the RD. The density inside the mask was subjected to a 3D classification, auto-refine and further postprocessing to obtain a good density map for next model building steps (Supplementary Fig. 5b). The maps were further subjected to sharpening using Relion post-processing^71^, LocScale^75^ and LocalDeblur^76^ (Supplementary Figs. 4h and 7h). The combination of all of them provided the best interpretability of the 3D reconstructed maps. For the difference map carried out between TH(DA) and apo-TH, the two maps were first normalized and filtered to the same resolution (that of the lowest one) and subtracted with the “vop subtract” option in the Chimera package.

For THNΔ35(DA) and THS40p, image processing was performed following similar steps and using Relion-3^77^ and cryoSPARC^78^. The 3D reconstruction process was carried out with D2 symmetry imposition. After several rounds of 2D and 3D classification, 152,128 and 148,453 particles were re-extracted at the original pixel size and used to generate the final 3D maps at 4.6 and 4.5 Å resolution, respectively.

### Model building, refinement and validation

First, the CD and OD domain structures from the human homotetrameric structure (PDB 2XSN) were fitted rigidly using Chimera^79^. Although a good fit in the Cryo-EM density map was obtained, a further flexible fitting step with iMODFIT^80^ was performed to optimize the arrangement of some flexible segments. The majority of the RD was modelled from the NMR structure of the rat homodimer (PDB 2MDA) with whom the human version shares 82% amino acid identity (Supplementary Fig. 2b), using the SWISS-MODEL homology-modelling server^81^. The resolution of the RD precluded accurate model building, but was sufficient to rigid-body fit the domain in a position compatible with its connection with the CD. Unfortunately, no homologous structure was found for the first 70 N-terminal amino acids of the RD. However, an un-modelled density was observed initially in the DA-bound structure that could accommodate a long α-helix structure. The secondary structure predictions obtained from I-TASSER^41^ and PSIPRED^40^ servers agreed in the existence of an approximately 20-residue long α-helix in this region (Supplementary Fig. 8a). Based on these observations, a generic 20-residue helix was modelled and fit into the corresponding density with Chimera. Then, we exhaustively scanned all possible orientations and sequence shifts for the first 70 residues of TH within this generic helix. The scanning included all 50 possible sequence shifts within a 20-residue window, as well as translations of the helix axis from −2 to +2 Å in 0.5 Å steps. The N-terminal α-helix configurations with lowest energy computed with KORP (knowledge-based orientational potential)^39^ corresponded to the 39–50 region, or to very close poses (i.e., slightly rotated and translated versions). Interestingly, the identified region corresponded to the fragment with highest α-helical propensity (Supplementary Fig. 8a). The minimum energy helix arrangement is proposed as the tentative backbone model. Supporting this model, the reconstruction lacking the first 35 residues (THNΔ35) in the presence of DA maintains an equivalent helix in the active site (Fig. 4a−c). Finally, the loops connecting the RD with the CD and the N-terminal helix were predicted with the RCD + server^82^. This online tool efficiently applies the RCD loop-closure algorithm^83^ to generate feasible loop conformations that are refined and ranked in PyRosetta 3.0^84^. The best loop conformations were selected to illustrate the feasibility of the connection.

The final TH models (Supplementary Fig. 5c, d) were subjected to a double real-space refinement, first manually using COOT^85^ and then, an automatic procedure with PHENIX 1-19-4092-000^86^ or REFMAC 5.8.0258^87^ implemented in the CCP-EM software platform^88^. The restraints used in the real-space refinement were both standard (bond, angle, planarity, chirality, dihedral, and non-bonded repulsion), and with some additional restraints (Ramachandran plot, C-beta deviations, rotamer, and secondary structure). A local grid search-based fit was included in the refinement strategy to resolve side-chain outliers (rotamers or poor map fitting). Several rounds of real-space refinement were performed until a stable final model was obtained and subsequently validated. For both apo-TH and TH(DA), several comparable Cryo-EM maps were used to combine information and refine the tracing of the whole atomic structure. Validation of the final models was done using the phenix_validation_Cryo-EM module in PHENIX. The figures with maps and structures have been rendered with PyMOL 2.2.0 and Chimera 1.14.

### Molecular dynamics (MD) simulations

A total of 8 all-atom unbiased MD simulations of tetrameric TH were carried out for 0.5 µs each. Simulations were performed with and without DA coordinated to the active site iron, as well as with and without phosphorylation of S40 (THS40p), for a total of four individual systems. The atomic tetramer models were based on the Cryo-EM structure of TH(DA) by inserting DA with the same orientation and iron-oxygen bonding distances as that of human PAH (PDB 5PAH), and by adding a phosphate group to S40. To enhance sampling and statistics, each state was simulated in parallel, differing in their generated random initial velocities. All atomic models were prepared with Amber 18 and the corresponding Amber14SB forcefield^89^. Parameters for DA as well as iron with coordinating residues were prepared with Antechamber^70^ and the general Amber forcefield^90^ using a semi-empirical model. Protonation states of side chains were assigned based on the 3D-structure using PROPKA at pH 7.0^86^. For each of the simulations, the system was neutralized using a mixture of Cl^−^ and Na^+^ counter ions, and the protein was solvated in a periodic truncated octahedron box with TIP3 water molecules^91^, providing 16 Å of water between the protein surface and the periodic box edge. The solute was minimized for 10,000 steps, followed by 10,000 steps of minimization of the whole system with restraints on the protein backbone, and finally 20,000 steps of minimization of all atoms. The protein was then heated to 100 K with weak restraints for 20 ps, and to 300 K for 1 ns. Equilibration with constant pressure and temperature (NPT) of the system was performed for a total of 20 ns prior to the production with reduced restraints on the solute. The production runs lasted 500 ns and were performed at a constant volume and energy. All simulations were run with a 2 fs time step, using SHAKE constraints on bonds involving hydrogens. All simulations were run with GPU acceleration^92^ on Nvidia RTX 2080Ti cards, producing on average 48 ns of molecular dynamics per day of the systems simulated. The simulations were analyzed using cpptraj^93^ (AmberTools20). Distances, clustering and fluctuation profiles were shown at the monomer level, averaged over the 4 subunits from two simulations. The K-means algorithm was used for clustering and the conformation representing the largest cluster over the last 50 ns was selected to represent each system.

### Statistics and reproducibility

Statistical analyses were performed with Graphpad Prism software version 8.3.0. For SRCD, DSC and TH activity measurements, three enzyme samples from independent protein purifications were measured and the mean ± SD (standard deviation) was calculated. The homogeneity of variances was confirmed by Brown-Forsythe tests and multiple comparisons were made using one-way analysis of variance (ANOVA) followed by a post hoc HSD Tukey test. Differences in secondary structure content, thermal onset (*T*_onset_) and temperature maximum (*T*_max_) and half-maximal inhibitory concentration (IC50) of the individual TH forms when compared to themselves with/without DA and to full-length TH in the same state were considered significant when *p* *<* 0.05. The individual curves were fitted to a four-parameter logistic curve by the *[Inhibitor] vs. response—Variable slope* equation (*y* = min + ((max − min)/(1 + IC50/X)^Hill Slope) in Graphpad and the mean IC50 ± SD reported.

The results in the representative SDS-gel (Supplemental Fig. 3a) and Cryo-EM micrograph (Supplemental Fig. 3c) for purified apo-TH have been observed with at least three protein preparations in independent experiments.

### Reporting summary

Further information on research design is available in the Nature Research Reporting Summary linked to this article.

## Supplementary information

Supplementary information

Description of Additional Supplementary Files

Supplementary Movie 1

Reporting Summary

## Data Availability

The data that support this study are available from the corresponding authors upon reasonable request. Cryo-EM data have been deposited in the Electron Microscopy Data Bank under accession codes EMD-11624 for full length apo-TH, EMD-11467 for full length TH(DA), EMD-11587 for CD + OD domains of apo-TH, EMD-11309 for CD + OD domains of TH(DA) and EMD-13442 for THNΔ35. The associated atomic models have been deposited in the Protein Data Bank under accession codes 7A2G for full length apo-TH, 6ZVP for full length TH(DA), 6ZZU for apo-TH, CD + OD domains, 6ZN2 for TH(DA), CD + OD domains and 7PIM for THNΔ35. Previously reported structures used in this work are 1TOH, 2XSN, 2MDA, 5PAH, 1KW0, 6HYC. The mass spectrometry proteomics data have been deposited to the ProteomeXchange Consortium via the PRIDE partner repository with the dataset identifier PXD024519. Source data are provided with this paper.

## Source data

Source Data
