# Supplementary material for: Structural mechanism for tyrosine hydroxylase inhibition by dopamine and reactivation by Ser40 phosphorylation
Source: Nat Commun. 2022 Jan 10;13:74. doi: 10.1038/s41467-021-27657-y (PMC8748767; doi:10.1038/s41467-021-27657-y)
Supplement: Supplementary file 1 — Supplementary information [file 41467_2021_27657_MOESM1_ESM.pdf]

Supplementary Information

Structural mechanism for tyrosine hydroxylase inhibition by dopamine and reactivation by Ser40 phosphorylation

Maria Teresa Bueno-Carrasco[1#], Jorge Cuéllar[1#*], Marte I. Flydal[2#], César Santiago[1], Trond-André Kråkenes[2], Rune Kleppe[3], Jose R. López-Blanco[4], Knut Teigen[2], Sara Alvira[1,5], Pablo Chacón[4], Aurora Martinez[2*] and José M. Valpuesta[1*].

[1]Centro Nacional de Biotecnología (CNB-CSIC), Madrid, Spain

[2]Department of Biomedicine, University of Bergen, Bergen, Norway

[3]Norwegian Centre for Maritime and Diving Medicine, Department of Occupational Medicine, Haukeland University Hospital, Bergen, Norway

[4]Instituto de Química Física Rocasolano (IQFR-CSIC), Madrid, Spain

[5]Current address: School of Biochemistry, University of Bristol, BS8 1TD, United Kingdom

[#]These authors contributed equally to the work

*To whom correspondence should be addressed

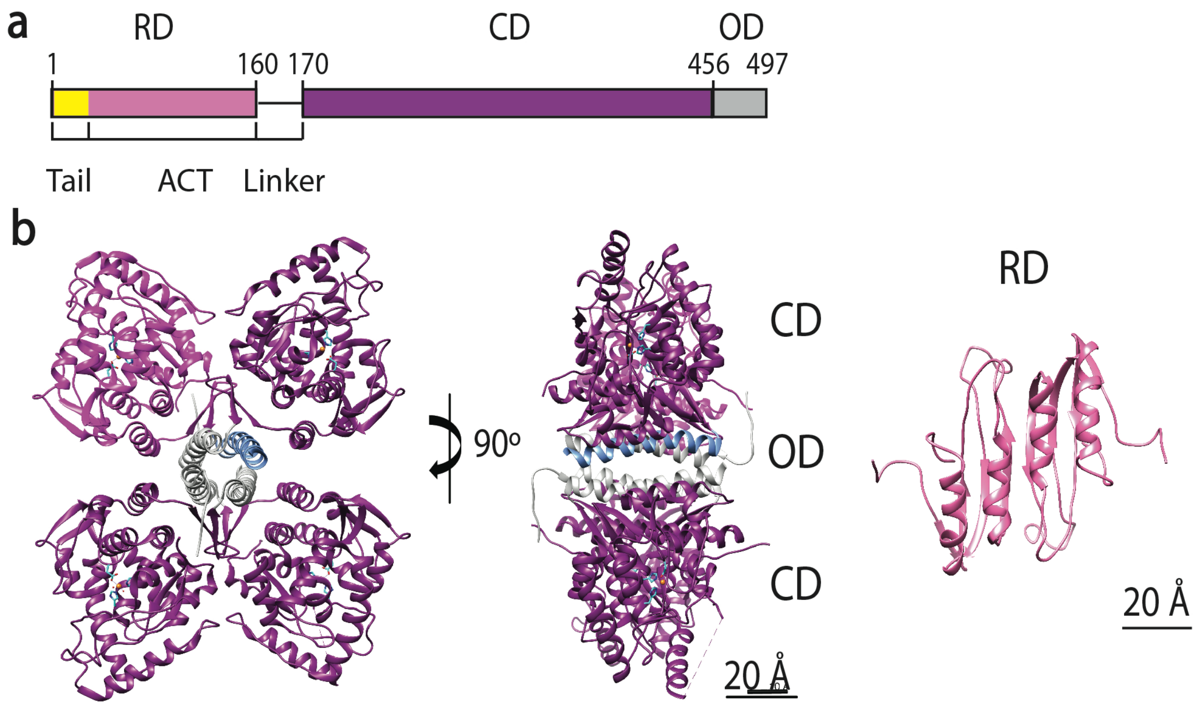

Supplementary Figure 1. Structural organization of tyrosine hydroxylase (TH). (a) Schematic arrangement of human TH showing the different domains. The Regulatory Domain (RD) contains an unstructured tail in its first 70 residues (yellow) and an aspartate kinase-chorismate mutase-TyrA (ACT) domain (pink). The RD and the Catalytic Domain (CD; dark magenta) are connected through a linker. The C-terminal part is involved in OD oligomerization (the Oligomerization Domain, OD; grey). **(b)** Truncated atomic structures of the human tetramer of TH comprising the CD and the OD (PDB 2XSN). Colour code as in (a). One of the subunits forming the tetramer is highlighted in light magenta and blue. On the right side, the atomic structure of the rat RD, solved by NMR (PDB 2MDA).

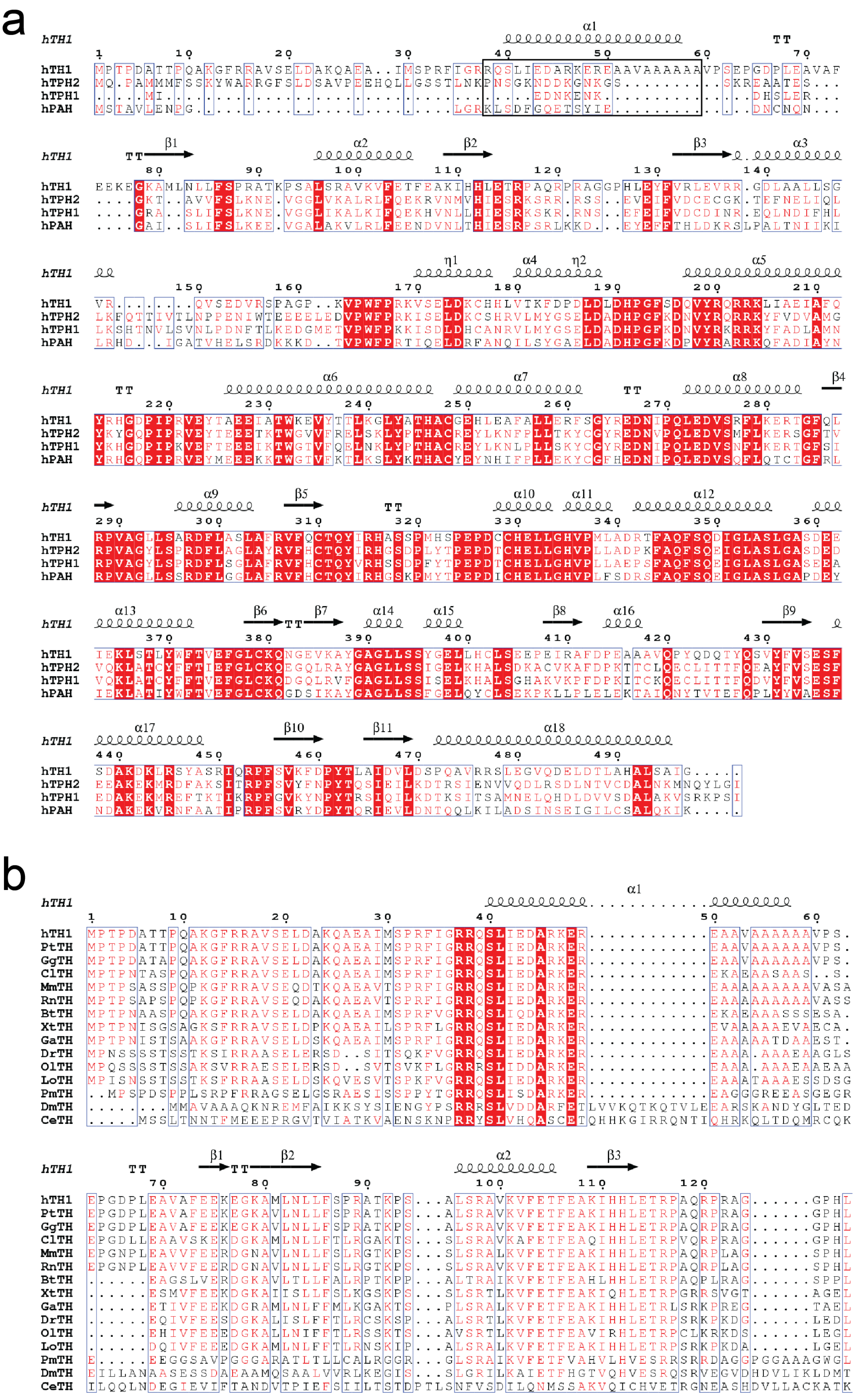
a
hTH1
hTH1
hTPH2
hTPH1
hPAH
b
hTH1
PtTH
GgTH
ClTH
MmTH
RnTH
BtTH
XtTH
GaTH
DrTH
OlTH
LoTH
PmTH
DmTH
CeTH

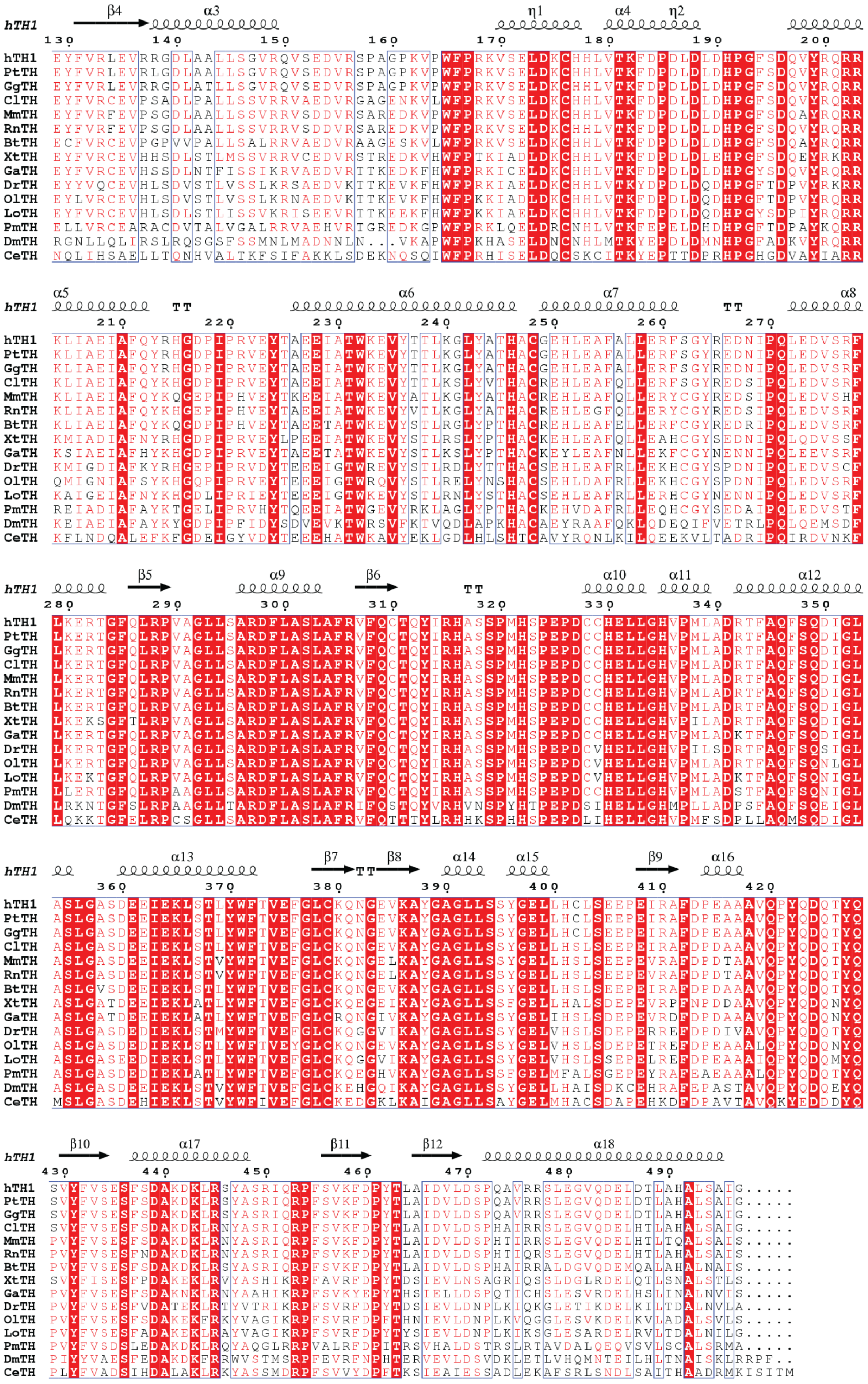

Supplementary Figure 2. Sequence alignment of members of the aromatic amino acid hydroxylase (AAAHs) family. (a) Alignment of the human AAAHs: hTH1 (tyrosine hydroxylase isoform 1; NP_000351.2), hPAH (phenylalanine hydroxylase; NP_000268.1), hTPH2 (tryptophan hydroxylase isoform 2; NP_775489.2), hTPH1 (tryptophan hydroxylase isoform 1; NP_004170.1). The area of alignment to the CD-interacting helix in hTH1 (residues 39-58) is marked by a black box. **(b)** Alignment of TH from selected organisms; h (*Homo sapiens* NP_000351.2), Pt (*Pan troglodytes*, XP_016775674.1), Gg (*Gorilla gorilla,*

XP_004050483.1), Cl (*Canis lupus*, NP_001002966.1), Mm (*Mus musculus*, NP_033403.1), Rn (*Rattus norvegicus*, NP_036872.1), Bt (*Bos taurus*, XP_024842736.1), Xt (*Xenopus tropicalis*, XP_031756384.1), Ga (*Gallus gallus*, NP_990136.1), Dr (*Danio rerio*, NP_571224.1), Ol (*Oryzias latipes*, NP_001265797.1), Lo (*Lepisosteus oculatus*, XP_006642630.1), Pm (*Petromyzon marinus*, XP_032805255.1), Dm (*Drosophila melanogaster*, NP_476897.1), Ce (*Caenorhabditis elegans*, NP_001254009.1). Secondary structure on top of the alignments is extracted from the TH(DA) structure (PDB 6ZVP). Alignments were carried out using Multialin and visualised in ESPript 3.0.

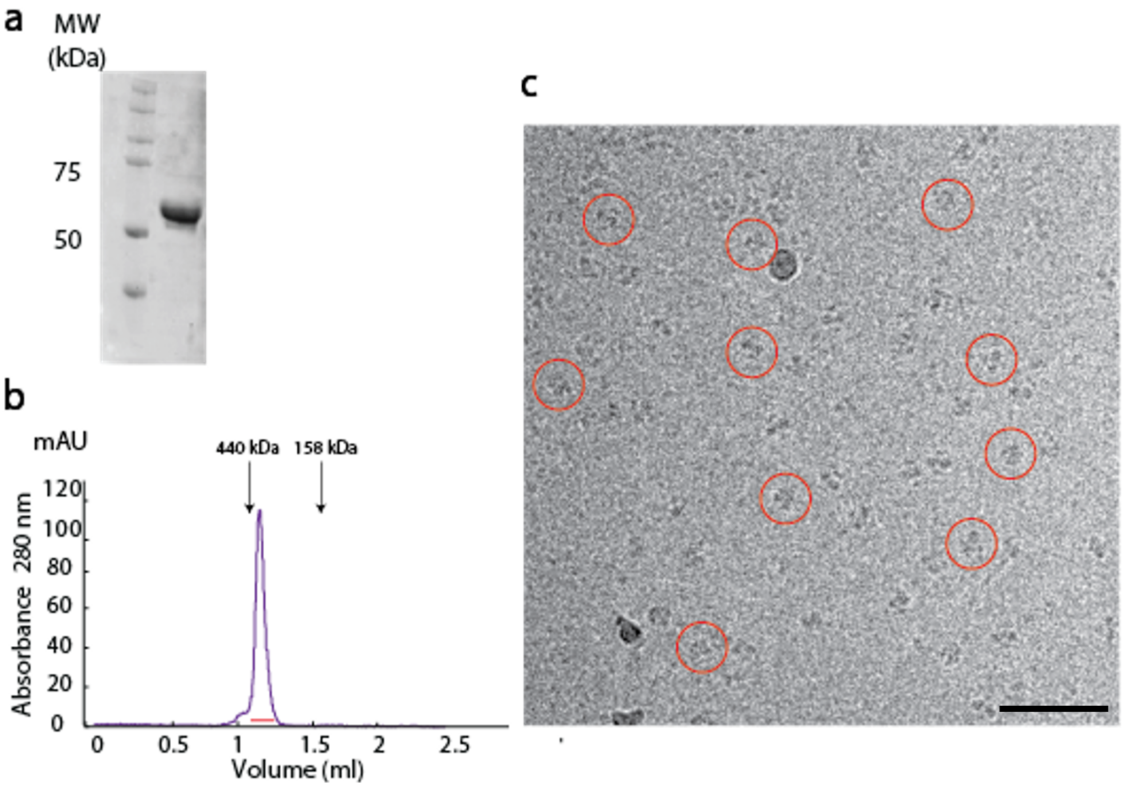

Supplementary Figure 3. Biochemical and EM analysis of TH. (a) SDS-PAGE analysis of the purity of apo-TH. The gel is representative from n>3 enzyme samples from independent protein purifications. **(b)** Size exclusion chromatography profile of apo-TH prior to vitrification. The red line highlights the fractions selected for further cryoEM analysis. **(c)** Representative cryoEM image of apo-TH, observed with n=3 enzyme samples from independent protein purifications. Particles in different orientations are marked within red circles. Bar indicates 500 Å.

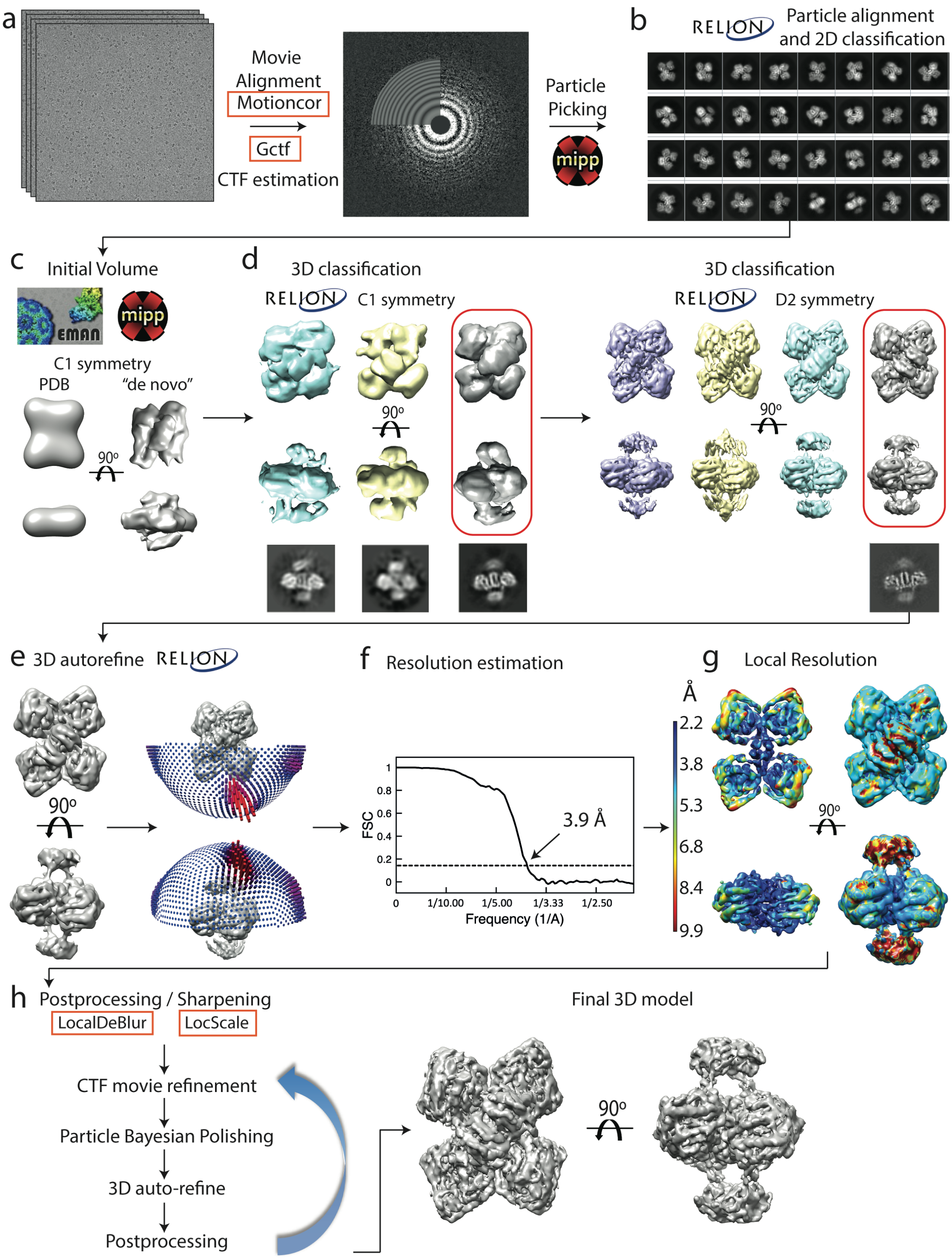

Supplementary Figure 4. Workflow of 3D reconstruction of apo-TH by cryoEM. (a) Movies were acquired in a 300 kV Titan Krios microscope. Alignment and Contrast Transfer Function (CTF) calculations were carried out to correct aberrations coming from the microscope. **(b)** 2D classification of the collected particles showing different orientations. **(c)** 3D reconstruction steps from an initial model (filtered at low resolution) without symmetry imposition**. (d)** Different 3D classifications (without and with D2 symmetry). **(e)** Refinement was performed to obtain the final map and the angular coverage. **(f)** Resolution estimation by the gold-standard criterion (FSC=0.143). **(g)** Estimation of local resolution. **(h)** Sharpening and polishing procedures were carried out to obtain the final 3D map for further atomic model building.

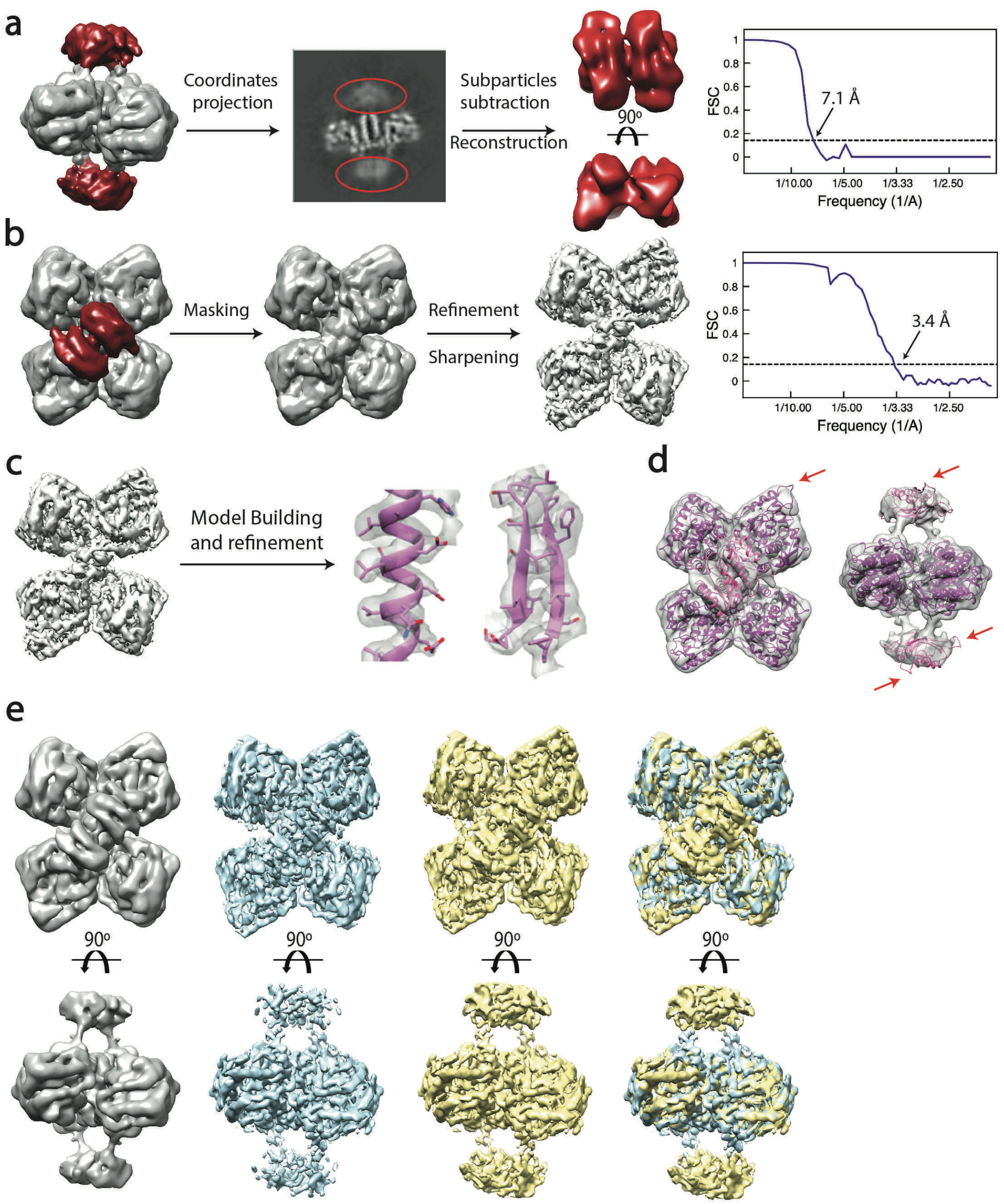

Supplementary Figure 5. Resolution improvement and model building. (a) Schematic steps in the local reconstruction process of the RD. **(b)** 3D reconstruction of the CD and OD after masking and post-processing steps. **(c)** The obtained 3D reconstruction of the CD+OD was used for model building. On the right some details of the definition of secondary structure elements. **(d)** Flexible fitting with iMODfit into the apo-TH refined map. Red arrows point to the more flexible loops that could not be fitted into the density map. **(e)** Sharpened density maps obtained using LocScale (yellow) and LocalDeBlur (blue) of the final map (grey). On the right, superimposition of the models obtained using both methods.

a

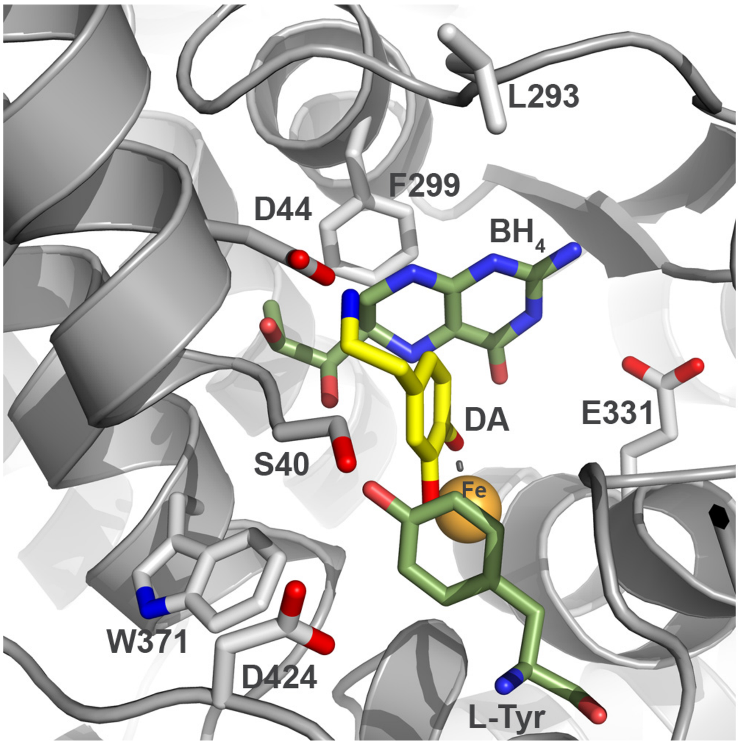

b

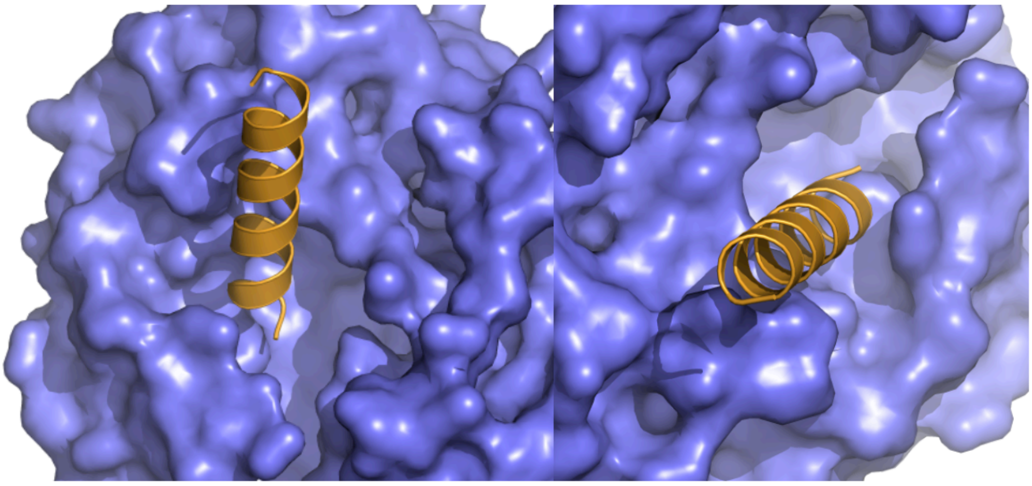

Supplementary Figure 6. (a) Docking of BH4 and L-tyrosine to TH(DA). L-tyrosine and BH4 were positioned in TH(DA) by alignment with structures of substrate and BH4-bound PAH (PDB 1KW0). BH4, L-tyrosine and DA are shown in coloured stick representation and interacting residues in TH(DA) as sticks with TH numbering. **(b)** Two views of the N-terminal α-helix (gold) inserted into the TH active site (surface representation). The images intend to show that there is ample space in the active site for for the disordered residues 1-39 to thread out of active site into the exterior.

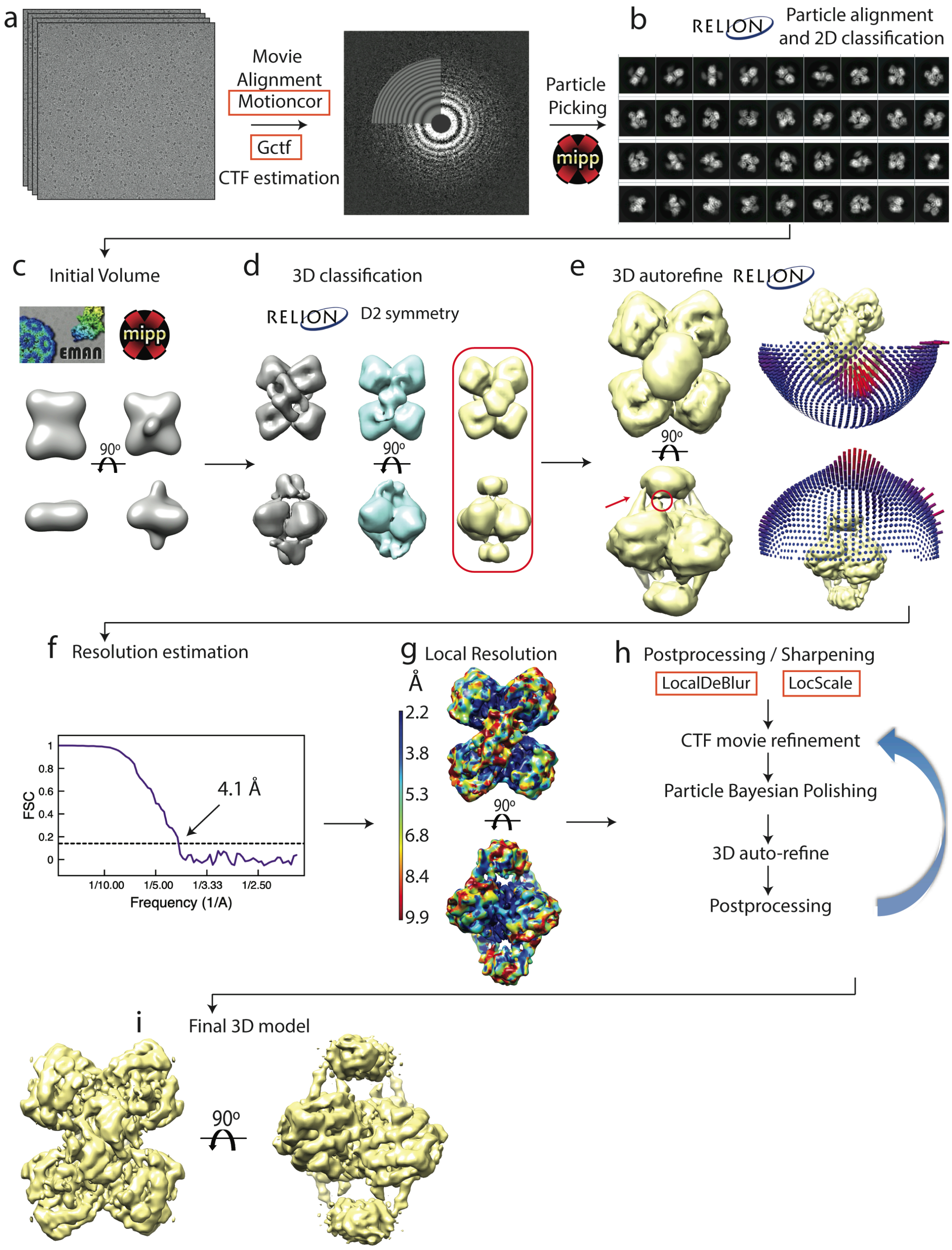

Supplementary Figure 7. Workflow of the 3D reconstruction of TH(DA) by cryoEM. (a) Movies were acquired in a 300 kV Titan Krios microscope. Alignment and CTF calculations were carried out to correct aberrations coming from the microscope. **(b)** 2D classification of the collected particles showing different orientations. **(c)** 3D reconstruction steps from an initial model (filtered at low resolution), without symmetry imposition. **(d)** 3D classification with D2 symmetry. **(e)** Refinement was performed to obtain the final map. **(f)** Resolution estimation by the gold-standard criterion (FSC= 0.143). **(g)** Estimation of local resolution. **(h)** Sharpening and polishing procedures were carried out to obtain **(i)** the final 3D map for further atomic model building.

a

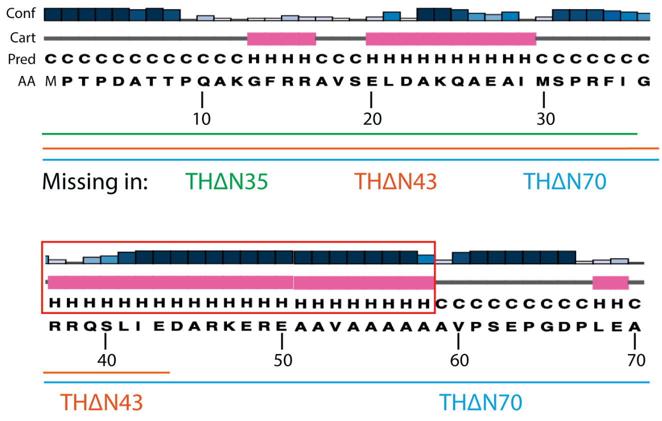

b

Apo-TH

Peptide 1				Peptide2			
Sequence	From	To	Linkage	Sequence	From	To	Linkage
[KER]	50	52	K1	{GAmGPTPDATTPQAK]	0	15	{0
[ATKPSALSR]	93	101	K3	{GAmGPTPDATTPQAK]	0	15	{0
{GAmGPTPDATTPQAK]	0	15	{0	[AVSELDAKQAEAImSPR]	20	36	K8
[ATKPSALSR]	93	101	T2	{GAmGPTPDATTPQAKGFRR]	0	19	{0
[SPAGPKVPWFPR]	160	171	K6	{GAmGPTPDATTPQAKGFRR]	0	19	{0
[SPAGPKVPWFPR]	160	171	K6	{GAmGPTPDATTPQAK]	0	15	T6
{GAmGPTPDATTPQAKGFRR]	0	19	{0	[RAVSELDAKQAEAImSPR]	19	36	K9
[ATKPSALSR]	93	101	T2	{GAmGPTPDATTPQAKGFRRAVSELDAKQAEAImSPR]	0	36	{0
[KER]	50	52	K1	{GAmGPTPDATTPQAKGFRR]	0	19	{0
[KLIAEIAFQYR]	207	217	K1	{GAmGPTPDATTPQAK]	0	15	{0
[AVKVFETFEAK]	102	112	K3	{GAmGPTPDATTPQAK]	0	15	{0
[FLKER]	281	285	K3	{GAmGPTPDATTPQAK]	0	15	{0
[DKLR]	445	448	K2	{GAmGPTPDATTPQAK]	0	15	{0
{GAMGPTPDATTPQAKGFRR]	0	19	{0	{GAmGPTPDATTPQAKGFRR]	0	19	{0
[KER]	50	52	K1	{GAmGPTPDATTPQAKGFRRAVSELDAKQAEAImSPR]	0	36	{0
{GAmGPTPDATTPQAK]	0	15	{0	[EGKAmLNLLFSPR]	80	92	K3
{GAmGPTPDATTPQAK]	0	15	{0	[RKLIAEIAFQYR]	206	217	K2
[DKLR]	445	448	K2	{GAmGPTPDATTPQAKGFRR]	0	19	{0
[SYASR]	449	453	S1	{GAmGPTPDATTPQAK]	0	15	{0

TH(DA)

Peptide 1				Peptide2			
Sequence	From	To	Linkage	Sequence	From	To	Linkage
[KER]	50	52	K1	{GAmGPTPDATTPQAK]	0	15	{0
[ATKPSALSR]	93	101	K3	{GAmGPTPDATTPQAK]	0	15	{0
[KER]	50	52	K1	{GAmGPTPDATTPQAKGFRR]	0	19	{0
[ATKPSALSR]	93	101	T2	{GAmGPTPDATTPQAKGFRR]	0	19	{0
[ATKPSALSR]	93	101	T2	{GAmGPTPDATTPQAKGFRRAVSELDAKQAEAImSPR]	0	36	{0
[SPAGPKVPWFPR]	160	171	K6	{GAmGPTPDATTPQAK]	0	15	T6
{GAmGPTPDATTPQAKGFRR]	0	19	{0	[RAVSELDAKQAEAImSPR]	19	36	K9
[SPAGPKVPWFPR]	160	171	K6	{GAmGPTPDATTPQAKGFRR]	0	19	{0
{GAmGPTPDATTPQAK]	0	15	{0	[AVSELDAKQAEAImSPR]	20	36	S3
[DKLR]	445	448	K2	{GAmGPTPDATTPQAK]	0	15	{0

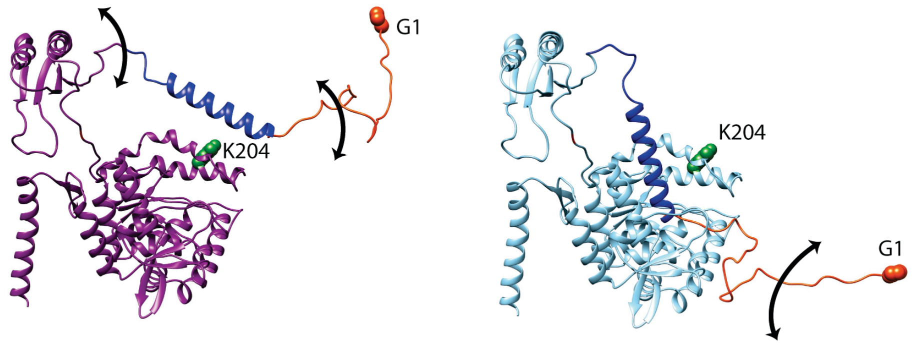

c

CROSSLINKED PEPTIDES		m/z	z	Peak Area	
Peptide 1	Peptide 2			APO-TH	TH(DA)
.**G1**PTPDATTPQAK.G	R.**K204**LIAEIAFQYR.H	983.18954	3+	$4.45 \cdot 10^5$	$9.88 \cdot 10^4$
.**G1**PTPDATTPQAK.G	R.R**K204**LIAEIAFQYR.H	776.67109	4+	$1.87 \cdot 10^5$	$6.56 \cdot 10^4$

d

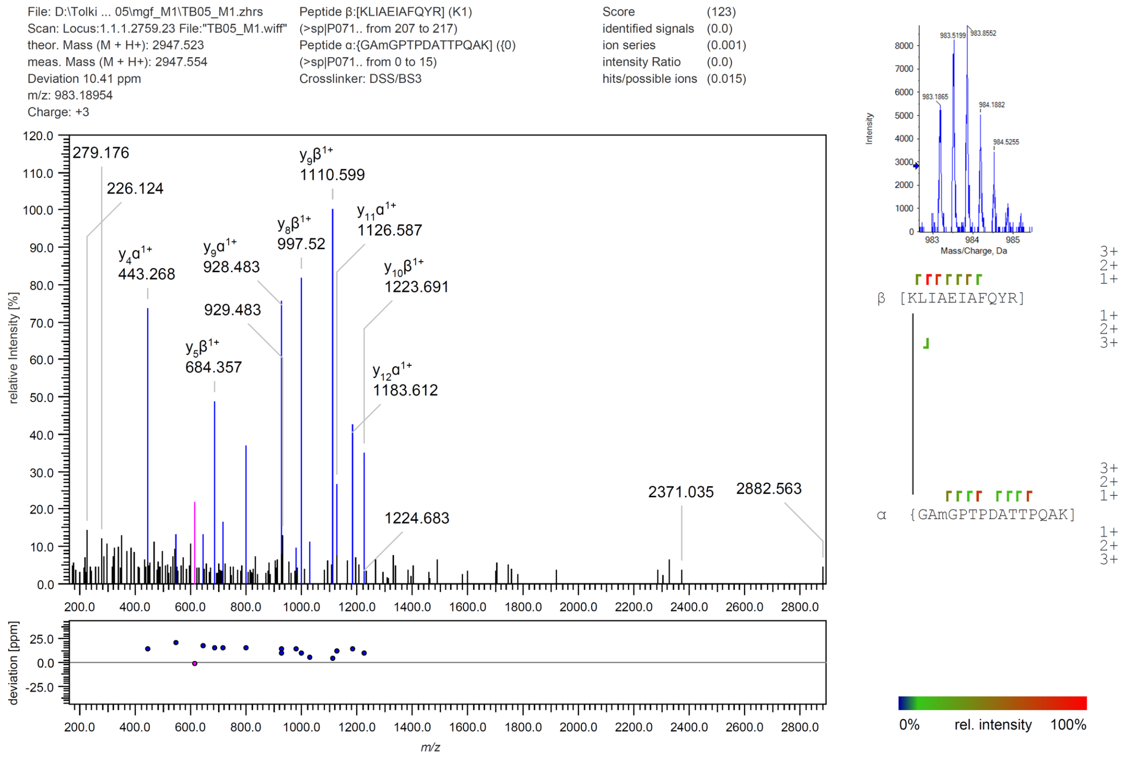

e

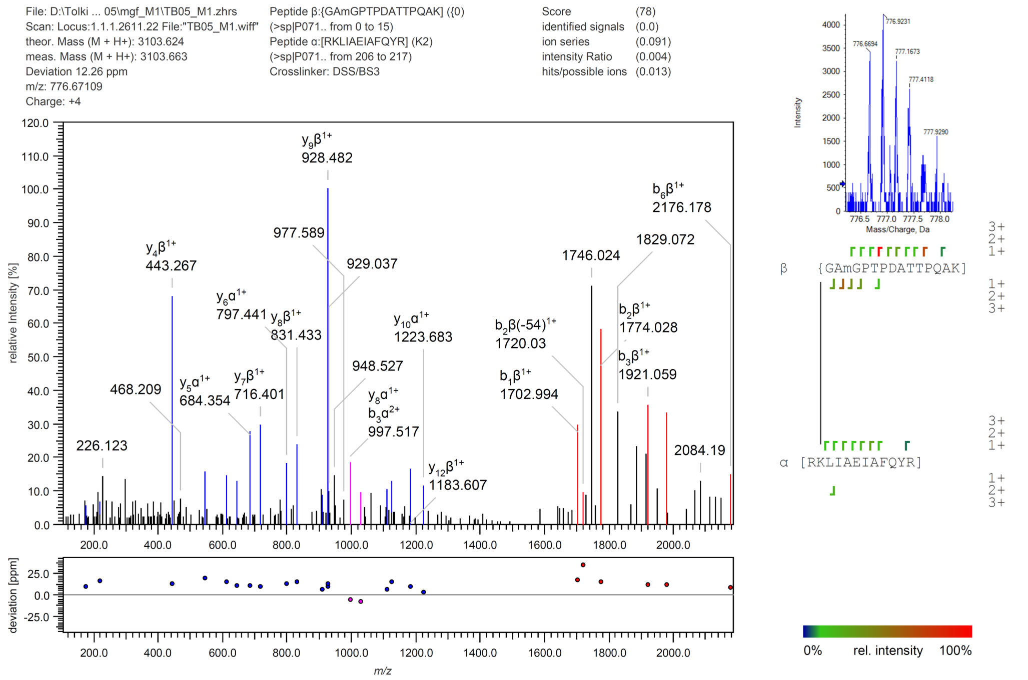

Supplementary Figure 8. Flexibility of the TH N-terminal tail. (a) Secondary structure prediction by PSIPRED of the first 70 residues of TH. The rows represent the confidence score,

with higher and darker bars representing improved prediction (Conf), the assignment in cartoon form (Cart), the assignment in letters H for helix and C for coil (Pred), and the query sequence (AA). The α-helices predicted are highlighted in pink. The red square marks the residues included in our predicted atomic model of TH(DA), which matches the highest confidence helix prediction by PSIPRED. The stretch of amino acids missing in the three truncated mutants are shown by lines in green (THNΔ35, lacking the first 35 residues), orange (THNΔ43, lacking the first 43 residues) and blue (THNΔ70, lacking the whole disordered tail), respectively. **(b)** Upper panel: XL-MS analysis of apo-TH and TH(DA). The identified peptides involving the first 20 N-terminal residues of the protein are shown. The sequence, position of the first and last residue of each peptide and the crosslinking site are indicated. Lower panel: a model of the proposed rearrangement of the N-terminal region of TH upon DA binding based on the XL-MS data. On the left, the atomic model of an apo-TH monomer showing a modelled N-terminal tail. The black arrows show the two hinge regions that confer flexibility to the N-terminal part. On the right, the atomic model of TH(DA) monomer showing just one hinge point as the α-helix is fixed in the active site. The stabilization of the helix reduces the flexibility of the unstructured N-terminal domain and the possibility of crosslinking reactions. The lysine K204 located in the CD is highlighted in green, whereas the free amine group located at the N-terminal is coloured orange. **(c)** Crosslinks between the protein N-terminus and K204. The sequence of both peptides, their m/z ratios, charge and peak areas in the Apo-TH and TH(Da) samples are indicated. The residues involved in the crosslink are depicted in red. The residues before and after the trypsin cleavage site are indicated. **(d and e)** Annotated MS2 spectra of the peptides displayed in (c). Signals assigned to ions of the y and b series are represented in blue and red, respectively. Signals that may be assigned to more than one ion series are represented in magenta. The upper right panel shows the isotopic envelope of the corresponding precursor ion

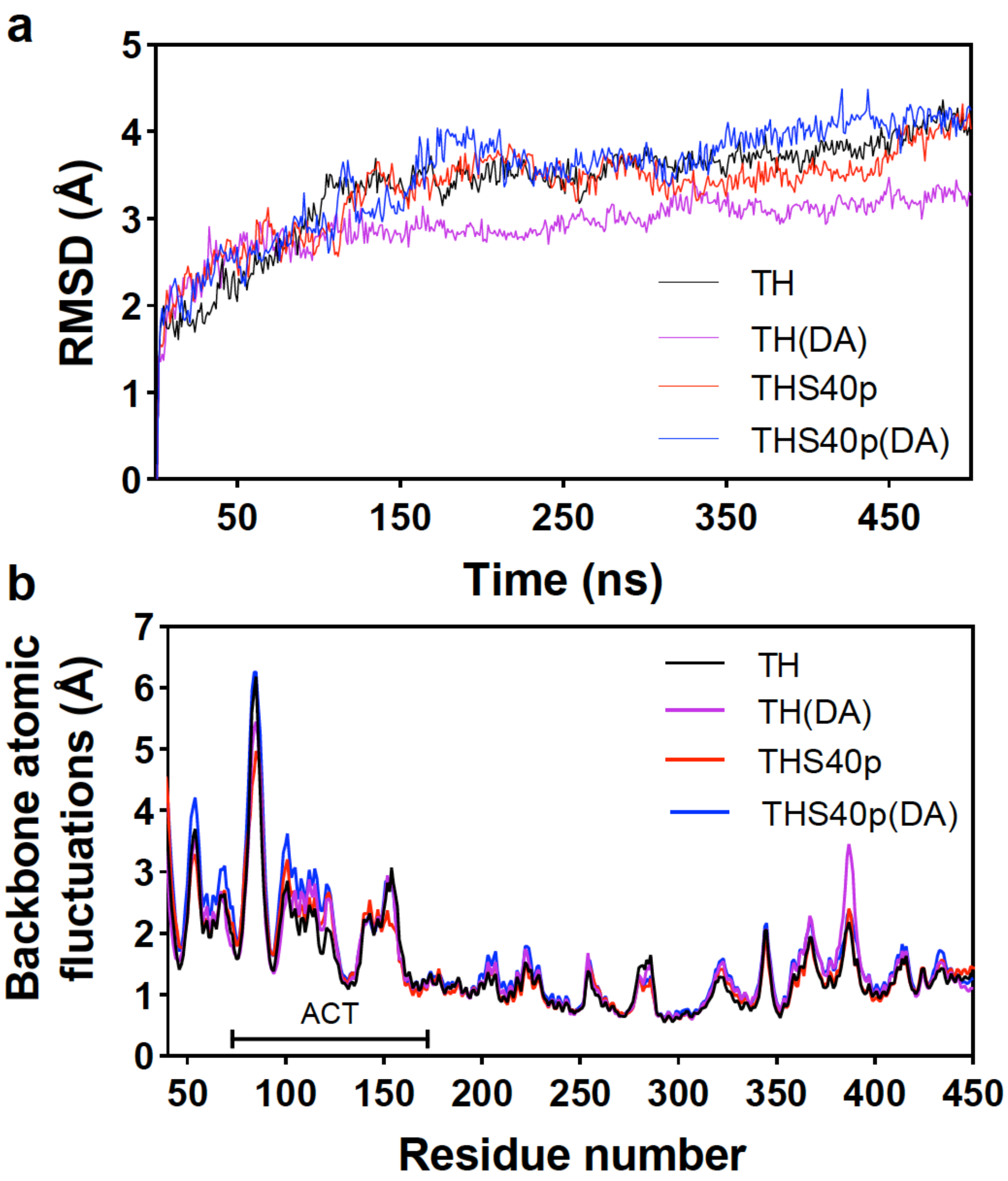

Supplementary Figure 9. Root-mean-square deviation and fluctuation plot for the MD simulations. (a) Backbone RMSD versus time during the first 450 ns MD simulations of TH (green), TH(DA) (magenta), THS40p (red) and THS40p(DA) (blue). The RMSD of Cα positions were calculated from superimposed trajectory frames of all four individual systems as a function of simulation time. **(b)** Backbone atomic positional fluctuations of all four simulated systems as a function of residue number for the 4 simulated systems. Fluctuations were averaged over 8 monomers for each system (2 tetramers simulated in parallel). Source data are provided as a Source Data file.

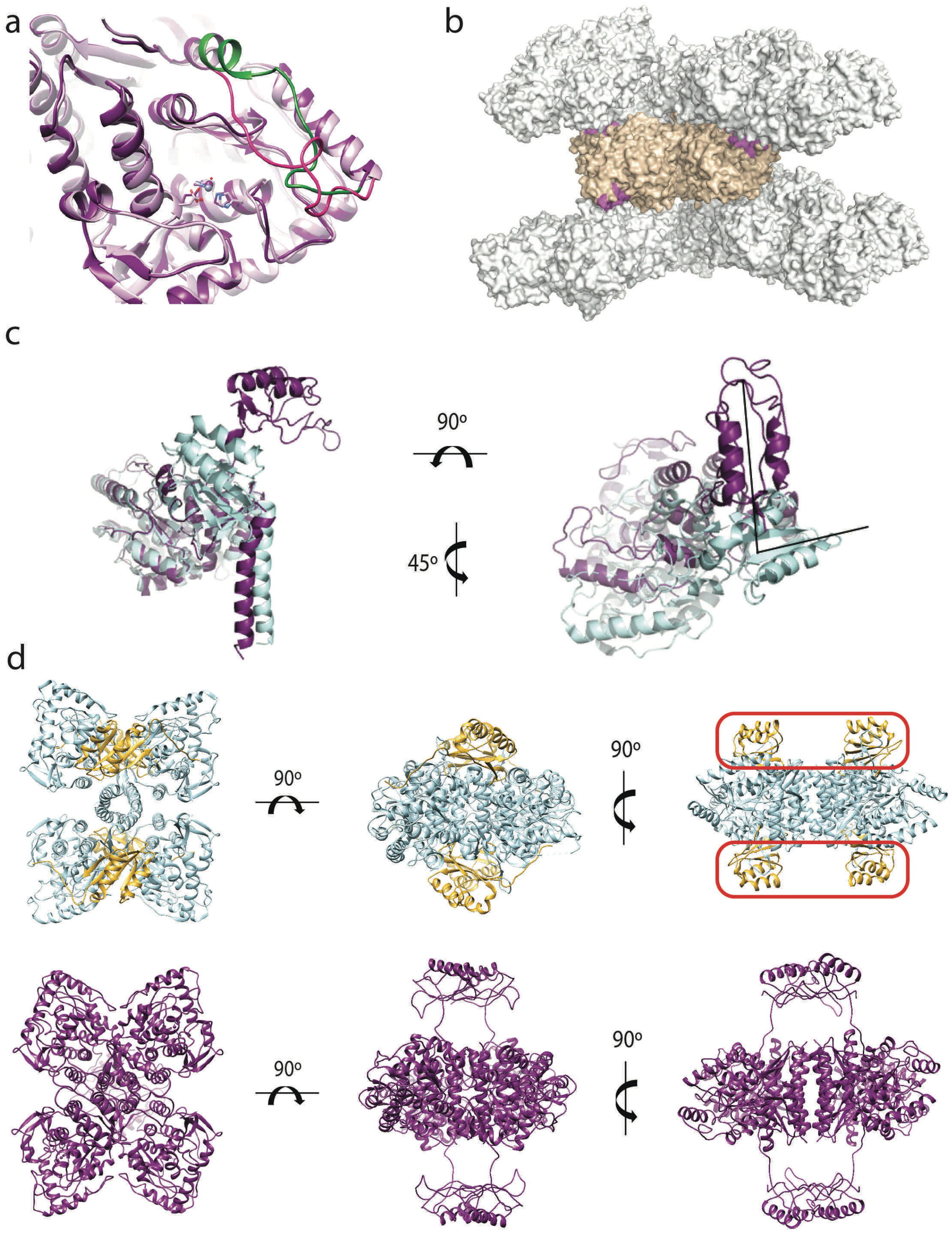

Supplementary Figure 10. Structural comparisons. (a) Structural differences in the 176-196 segment of the active site between the crystallographic TH (red; PDB 2XSN)) and that obtained using cryoEM (green) structures. **(b)** Crystal arrangement of TH which shows that the segment described in (a) is involved in crystal packing. **(c)** Two orthogonal views of the atomic models of the monomer of PAH (cyan) and TH (dark magenta) in which the CD has been used to align the two structures, showing (left) the tilt that takes place between the two OD helices (12.7°) (the lines depict the axes of the two OD helices), and (right) the angular shift between the two RDs (88°) (the lines depict the longitudinal axes of the two RDs). **(d)** The same three orthogonal views of the atomic models of PAH (PDB 6HYC), with the CD+OD in light blue and the RD in gold (upper panels), and TH (this work; lower panels).

a

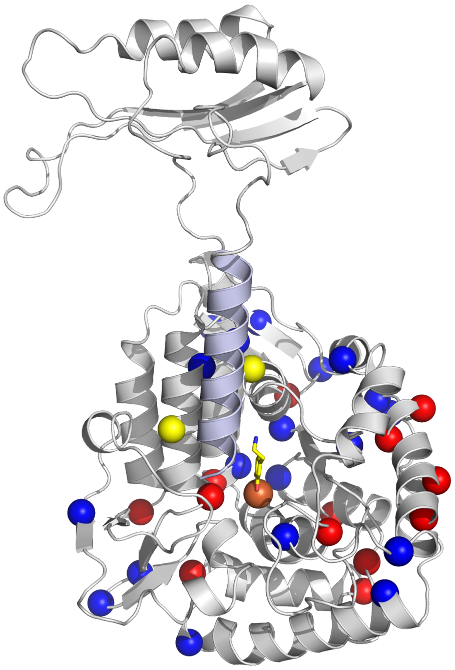

b

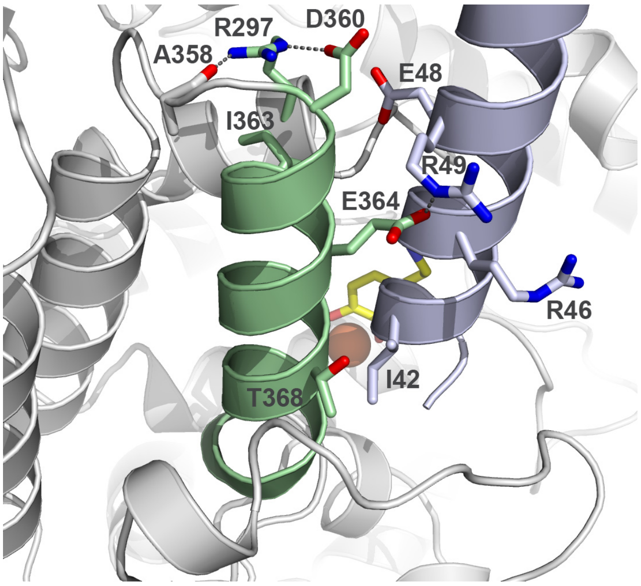

Supplementary Figure 11. The location of THD-associated missense mutations in the TH structure. (a) Residues with THD mutations registered in the PND database (http://www.biopku.org/pnddb) are shown in the subunit structure of TH(DA) as spheres representing Cα-atom coloured blue for type A mutations and red for type B mutations, except for R297W and T368M, which are shown in yellow. **(b)** A closer view of the interaction networks established by residues R297 and T368. These interactions are expected to be disrupted in TH mutants R297W and T368M associated with large destabilization. Interacting residues and DA are shown as sticks, iron as an orange sphere. The helices 39-58 and 360-375 are shown in blue and green, respectively.

Data collection	Apo-TH	TH(DA)
**Microscope**	Titan Krios	Titan Krios
**Voltage (keV)**	300	300
**Detector**	K2 Summit	K2 Summit
**Nominal magnification**	130,000x	130,000x
**Pixel size (Å)**	1.047	1.053
**Defocus range (µm)**	-1.6 to -2.2	-1.8 to -3.2
**Exposure time (s)**	9	6
**Electron dose (e^-/Å^2)**	39.6	37
**Frames**	36	40
**Dose/frame (e^-/Å^2)**	1.1	0.925
**Movies (no.)**	3867	4422
**Initial particles (no.)**	1,244,162	1,550,655
**Final particles (no.)**	29,418	36,368
**Final resolution (Å)**	Unmasked / Masked	Unmasked / Masked
	4.5 / 4.2	4.3 / 4.1

Supplementary Table 1. Data collection parameters of apo-TH and TH(DA).

Model	Apo-TH short	
**Composition (#)**		
**Chains**	8	
**Atoms**	10760 (Hydrogens: 0)	
**Residues**	Protein: 1340 Nucleotide: 0	
**Water**	4	
**Ligands**	FE: 4	
**Bonds (RMSD)**		
**Length (Å) (# > 4σ)**	0.006 (0)	
**Angles (°) (# > 4σ)**	1.211 (3)	
**MolProbity score**	1.87	
**Clash score**	10.32	
**Ramachandran plot (%)**		
**Outliers**	0.00	
**Allowed**	4.88	
**Favored**	95.12	
**Rotamer outliers (%)**	0.17	
**Cβ outliers (%)**	0.00	
**Peptide plane (%)**		
**Cis proline/general**	0.0/0.0	
**Twisted proline/general**	0.0/0.0	
**CaBLAM outliers (%)**	1.51	
**ADP (B-factors)**		
**Iso/Aniso (#)**	10760/0	
**min/max/mean**		
**Protein**	102.60/158.44/122.30	
**Nucleotide**	---	
**Ligand**	145.89/160.59/151.98	
**Water**	117.16/131.59/126.52	
**Occupancy**		
**Mean**	1.00	
**occ = 1 (%)**	100.00	
**0 < occ < 1 (%)**	0.00	
**occ > 1 (%)**	0.00	
**Data**		
**Box**		
**Lengths (Å)**	99.75, 66.15, 119.70	
**Angles (°)**	90.00, 90.00, 90.00	
**Supplied Resolution (Å)**	3.5	
**Resolution Estimates (Å)**	Masked	Unmasked
**d FSC (half maps; 0.143)**	3.6	3.8
**d 99 (full/half1/half2)**	2.4/5.5/5.5	2.4/5.1/5.1
**d model**	2.0	2.0
**d FSC model (0/0.143/0.5)**	1.9/2.6/3.5	1.9/2.6/3.5
**Map min/max/mean**	-1.27/1.54/0.01	
**Model vs. Data**		
**CC (mask)**	0.78	
**CC (box)**	0.79	
**CC (peaks)**	0.79	
**CC (volume)**	0.78	
**Mean CC for ligands**	0.84	

Supplementary Table 2. Apo-TH model refinement and statistics of the CD and OD domains.

Model	Apo-TH full-length	
**Composition (#)**		
**Chains**	8	
**Atoms**	13408 (Hydrogens: 0)	
**Residues**	Protein: 1680 Nucleotide: 0	
**Water**	4	
**Ligands**	FE: 4	
**Bonds (RMSD)**		
**Length (Å) (# > 4σ)**	0.006 (0)	
**Angles (°) (# > 4σ)**	0.846 (2)	
**MolProbity score**	2.83	
**Clash score**	46.31	
**Ramachandran plot (%)**		
**Outliers**	0.24	
**Allowed**	16.33	
**Favored**	83.43	
**Rotamer outliers (%)**	0.49	
**Cβ outliers (%)**	0.00	
**Peptide plane (%)**		
**Cis proline/general**	0.0/0.0	
**Twisted proline/general**	4.0/0.2	
**CaBLAM outliers (%)**	4.45	
**ADP (B-factors)**		
**Iso/Aniso (#)**	13408/0	
**min/max/mean**		
**Protein**	199.65/541.25/325.31	
**Nucleotide**	---	
**Ligand**	287.16/310.30/297.41	
**Water**	266.14/277.67/271.84	
**Occupancy**		
**Mean**	1.00	
**occ = 1 (%)**	100.00	
**0 < occ < 1 (%)**	0.00	
**occ > 1 (%)**	0.00	
**Data**		
**Box**		
**Lengths (Å)**	101.56, 118.31, 128.78	
**Angles (°)**	90.00, 90.00, 90.00	
**Supplied Resolution (Å)**	4.2	
**Resolution Estimates (Å)**	Masked	Unmasked
**d FSC (half maps; 0.143)**	4.2	4.5
**d 99 (full/half1/half2)**	6.4/2.1/2.1	6.2/2.1/2.1
**d model**	6.0	6.0
**d FSC model (0/0.143/0.5)**	3.6/4.2/5.9	3.6/4.3/6.1
**Map min/max/mean**	-0.39/0.74/0.01	
**Model vs. Data**		
**CC (mask)**	0.80	
**CC (box)**	0.86	
**CC (peaks)**	0.72	
**CC (volume)**	0.81	
**Mean CC for ligands**	0.84	

Supplementary Table 3. Apo-TH model refinement and statistics of the full-length protein.

Model	TH(DA) short	
**Composition (#)**		
Chains	8	
Atoms	11320 (Hydrogens: 0)	
Residues	Protein: 1412 Nucleotide: 0	
Water	0	
Ligands	LPD: 4 FE: 4	
**Bonds (RMSD)**		
Length (Å) (# > 4σ)	0.004 (0)	
Angles (°) (# > 4σ)	0.921 (20)	
**MolProbity score**	2.75	
**Clash score**	22.57	
**Ramachandran plot (%)**		
Outliers	0.00	
Allowed	4.30	
Favored	95.70	
**Rotamer outliers (%)**	6.35	
**Cβ outliers (%)**	0.00	
**Peptide plane (%)**		
Cis proline/general	0.0/0.0	
Twisted proline/general	0.0/0.0	
**CaBLAM outliers (%)**	1.74	
**ADP (B-factors)**		
Iso/Aniso (#)	11320/0	
min/max/mean		
Protein	44.92/440.00/259.30	
Nucleotide	---	
Ligand	132.58/404.81/271.04	
Water	---	
**Occupancy**		
Mean	0.98	
occ = 1 (%)	97.95	
0 < occ < 1 (%)	0.00	
occ > 1 (%)	0.00	
**Data**		
**Box**		
Lengths (Å)	105.30, 87.40, 122.15	
Angles (°)	90.00, 90.00, 90.00	
**Supplied Resolution (Å)**	4.5	
**Resolution Estimates (Å)**	Masked	Unmasked
d FSC (half maps; 0.143)	4.3	4.3
d 99 (full/half1/half2)	7.2/2.3/7.2	7.2/2.3/7.2
d model	6.5	6.5
d FSC model (0/0.143/0.5)	3.7/4.2/5.2	3.8/4.2/5.3
**Map min/max/mean**	-0.01/0.04/0.00	
**Model vs. Data**		
**CC (mask)**	0.85	
**CC (box)**	0.93	
**CC (peaks)**	0.82	
**CC (volume)**	0.85	
**Mean CC for ligands**	0.83	

Supplementary Table 4. TH(DA) model refinement and statistics of the CD and OD.

Model	TH(DA) full-length	
**Composition (#)**		
Chains	8	
Atoms	14552 (Hydrogens: 0)	
Residues	Protein: 1832 Nucleotide: 0	
Water	0	
Ligands	LPD: 4 FE: 4	
**Bonds (RMSD)**		
Length (Å) (# > 4σ)	0.002 (0)	
Angles (°) (# > 4σ)	0.600 (0)	
**MolProbity score**	2.72	
**Clash score**	18.98	
**Ramachandran plot (%)**		
Outliers	0.00	
Allowed	5.04	
Favored	94.96	
**Rotamer outliers (%)**	6.01	
**Cβ outliers (%)**	0.00	
**Peptide plane (%)**		
Cis proline/general	0.0/0.0	
Twisted proline/general	0.0/0.0	
**CaBLAM outliers (%)**	2.42	
**ADP (B-factors)**		
Iso/Aniso (#)	14552/0	
min/max/mean		
Protein	0.00/440.00/201.64	
Nucleotide	---	
Ligand	132.58/404.81/271.04	
Water	---	
**Occupancy**		
Mean	0.84	
occ = 1 (%)	84.36	
0 < occ < 1 (%)	0.00	
occ > 1 (%)	0.00	
**Data**		
**Box**		
Lengths (Å)	105.30, 130.57, 122.15	
Angles (°)	90.00, 90.00, 90.00	
**Supplied Resolution (Å)**	4.5	
**Resolution Estimates (Å)**	Masked	Unmasked
d FSC (half maps; 0.143)	4.0	4.0
d 99 (full/half1/half2)	6.4/4.7/6.4	5.2/4.7/5.1
d model	5.9	4.4
d FSC model (0/0.143/0.5)	4.2/5.1/7.0	4.2/5.2/7.0
**Map min/max/mean**	-0.01/0.04/0.00	
**Model vs. Data**		
**CC (mask)**	0.67	
**CC (box)**	0.84	
**CC (peaks)**	0.68	
**CC (volume)**	0.77	
**Mean CC for ligands**	0.85	

Supplementary Table 5. TH(DA) model refinement and statistics of the full-length protein.

Data collection	THNΔ35	THpS40
Microscope	Titan Krios	Titan Krios
Voltage (keV)	300	300
Detector	K3	K3
Nominal magnification	81,000x	81,000x
Pixel size (Å)	1.072	1.072
Defocus range (µm)	-1.4 to -3.4	-1.4 to -3.4
Exposure time (s)	2	2
Electron dose ($e^-/Å^2$)	30	30
Frames	40	40
Dose/frame ($e^-/Å^2$)	0.75	0.75
Movies (no.)	13213	9241
Initial particles (no.)	1,626,575	1,610,418
Final particles (no.)	152,128	148,453
Final resolution (Å)	Unmasked / Masked	Unmasked / Masked
	5.1 / 4.8	4.9 / 4.5

Supplementary Table 6. Data collection parameters of THNΔ35 and THS40p.

Model	THNΔ35(DA)	
**Composition (#)**		
Chains	8	
Atoms	11844 (Hydrogens: 524)	
Residues	Protein: 1412 Nucleotide: 0	
Water	0	
Ligands	LPD: 4 FE: 4	
**Bonds (RMSD)**		
Length (Å) (# > 4σ)	0.001 (0)	
Angles (°) (# > 4σ)	0.389 (0)	
**MolProbity score**	2.13	
**Clash score**	44.65	
**Ramachandran plot (%)**		
Outliers	0.00	
Allowed	2.01	
Favored	97.99	
**Rotamer outliers (%)**	0.67	
**Cβ outliers (%)**	0.00	
**Peptide plane (%)**		
Cis proline/general	0.0/0.0	
Twisted proline/general	0.0/0.0	
**CaBLAM outliers (%)**	0.87	
**ADP (B-factors)**		
Iso/Aniso (#)	11320/0	
min/max/mean		
Protein	164.74/408.43/274.54	
Nucleotide	---	
Ligand	274.45/331.72/280.01	
Water	---	
**Occupancy**		
Mean	0.95	
occ = 1 (%)	94.80	
0 < occ < 1 (%)	0.00	
occ > 1 (%)	0.00	
**Data**		
**Box**		
Lengths (Å)	103.98, 91.12, 121.14	
Angles (°)	90.00, 90.00, 90.00	
**Supplied Resolution (Å)**	4.5	
**Resolution Estimates (Å)**	Masked	Unmasked
d FSC (half maps; 0.143)	4.8	5.1
d 99 (full/half1/half2)	5.7/8.4/8.2	5.6/8.1/7.9
d model	6.0	6.0
d FSC model (0/0.143/0.5)	3.5/4.4/6.2	3.5/4.4/6.2
**Map min/max/mean**	-0.03/0.06/0.00	
**Model vs. Data**		
**CC (mask)**	0.81	
**CC (box)**	0.86	
**CC (peaks)**	0.78	
**CC (volume)**	0.82	
**Mean CC for ligands**	0.80	

Supplementary Table 7. THND35(DA) model refinement and statistics of the full-length protein.

	α-helices (%)		β-sheets (%)		Turns (%)		Others (%)	
DA	-	+	-	+	-	+	-	+
**TH**	31.5 ± 1.4	30.2 ± 0.9	15.3 ± 6.2	19.5 ± 1.0	15.3 ± 2.0	13.5 ± 1.1	38.0 ± 2.8	36.9 ± 1.0
**THNΔ35**	31.4 ± 1.0	32.2 ± 1.4	19.1 ± 1.6	18.2 ± 1.1	13.2 ± 1.0	13 ± 0.3	36.4 ± 0.6	36.5 ± 1.5
**THNΔ43**	29.2 ± 0.4	28 ± 0.4	19.5 ± 1.0	21.6 ± 2.5	13.5 ± 0.3	12.9 ± 0.7	37.9 ± 0.8	37.4 ± 1.6
**THNΔ70**	24.0 ± 2.9***	22.9 ± 2.5***	23.1 ± 3.5	25.6 ± 2.0	14.0 ± 0.2	14.1 ± 0.6	38.9 ± 0.8	37.5 ± 1.2
**THS40p**	27.6 ± 0.1	28.9 ± 1.4	21.8 ± 2.0	18.7 ± 1.3	13.6 ± 0.8	13.6 ± 0.3	37.0 ± 1.3	38.8 ± 2.3

Supplementary Table 8. Secondary structure content of TH and truncated mutants without and with DA. The values are presented as mean percentage ± SD, calculated in BestSel using far-UV spectra obtained with SRCD (Fig. 5a,b). Asterisks indicate statistically significant changes compared to wild-type TH in the same state using one-way ANOVA followed by Tukey's multiple comparisons test; p = 0.0002 (***) for THNΔ70 vs. TH., p = 0.0003 (***) for THNΔ70+DA vs TH+DA. DA did not induce a significant change in any of the TH forms. Number of samples (n) for each protein form = 3. Source data are provided as a Source Data file.

	T_{onset} (°C)		T_{max} (°C)	
DA	-	+	-	+
**TH**	43.3 ± 0.2	45.2 ± 0.6*(b)	54.9 ± 0.4	58.6 ± 0.6***(b)
**THNΔ35**	37.5 ± 1.2****(a)	39.6 ± 0.3****(a).**(b)	51.7 ± 0.7**(a)	54.9 ± 1.0***(a),***(b)
**THNΔ43**	43.3 ± 0.9	43.5 ± 0.5*(a)	54.3 ± 1.7	56.5 ± 0.4*(a),*(b)
**THNΔ70**	45.0 ± 0.5	45.6 ± 0.6	56.0 ± 0.1	56.4 ± 0.2

Supplementary Table 9. Effect of DA on the thermal stability of TH and truncated mutants. T_{onset} (taken as 5 percent of maximum heat capacity prior to the transition after normalization) and T_{max} are derived from the DSC thermograms. Asterisks indicate significant changes compared to TH in the same state (a) or to the same sample without DA (b) using one-way ANOVA followed by Tukey's multiple comparisons test; p (T_{onset}) = < 0.0001 (****) and p (T_{max}) = 0.0028 (**) for THNΔ35 vs. TH; p (T_{onset}) = < 0.0001 (****) and p (T_{max}) = 0.0002 (***) for THNΔ35+DA vs. TH+DA; p (T_{onset}) = < 0.0256 (*) and p (T_{max}) = 0.0321 (*) for THNΔ43+DA vs. TH+DA; p (T_{onset}) = < 0.0330 (*) and p (T_{max}) = 0.0004 (***) for TH+DA vs. TH; p (T_{onset}) = < 0.0063 (**) and p (T_{max}) = 0.0009 (***) for THNΔ35+DA vs. THNΔ35 and p (T_{max}) = 0.0176 (*) for THNΔ70+DA vs. THNΔ70. Source data and p-values are provided as a Source data file.

**Atom pair**	**TH(DA) (Å)**	**THS40p(DA) (Å)**	**TH (Å)**	**THS40p (Å)**
**Fe-S40(CA)**	9.7±0.1	10.1±0.1	10.7±0.2	11.1±0.2
**Fe-D44(CA)**	13.1±0.1	13.7±0.2	14.5±0.2	14.7±0.2
**Fe-D44(CG)**	11.3±0.2	12.1±0.2	13.3±0.3	13.6±0.2
**DA(N1)-D44(CA)**	6.9±0.2	7.6±0.3		
**DA(N1)-D44(CG)**	4.5±0.3	5.5±0.3		

Supplementary Table 10. Interatomic distances obtained from MD simulations. Average interatomic distances over the last 50 ns of simulation (450 ns to 500 ns) for selected atom pairs. Distances are an average of 8 individual measurements (4 subunits x 2 independent simulations).
