# Supplementary material for: Structural mechanism for tyrosine hydroxylase inhibition by dopamine and reactivation by Ser40 phosphorylation
Source: Nat Commun. 2022 Jan 10;13:74. doi: 10.1038/s41467-021-27657-y (PMC8748767; doi:10.1038/s41467-021-27657-y)
Supplement: Supplementary file 2 — Description of Additional Supplementary Files [file 41467_2021_27657_MOESM2_ESM.pdf]

Description of Additional Supplementary Files

File Name: Supplementary Movie 1
Description: Structural rearrangements upon DA binding
