# Supplementary material for: Structural mechanism for tyrosine hydroxylase inhibition by dopamine and reactivation by Ser40 phosphorylation
Source: Nat Commun. 2022 Jan 10;13:74. doi: 10.1038/s41467-021-27657-y (PMC8748767; doi:10.1038/s41467-021-27657-y)
Supplement: Supplementary file 4 — Reporting Summary [file 41467_2021_27657_MOESM4_ESM.pdf]

nature research

Corresponding author(s): Jorge Cuéllar, Aurora Martínez and José M. Valpuesta

Last updated by author(s): Oct 29, 2021

Reporting Summary

Nature Research wishes to improve the reproducibility of the work that we publish. This form provides structure for consistency and transparency in reporting. For further information on Nature Research policies, see our Editorial Policies and the Editorial Policy Checklist.

Statistics

For all statistical analyses, confirm that the following items are present in the figure legend, table legend, main text, or Methods section.

n/a	Confirmed	
☐	☒	The exact sample size (*n*) for each experimental group/condition, given as a discrete number and unit of measurement
☐	☒	A statement on whether measurements were taken from distinct samples or whether the same sample was measured repeatedly
☐	☒	The statistical test(s) used AND whether they are one- or two-sided<br>*Only common tests should be described solely by name; describe more complex techniques in the Methods section.*
☒	☐	A description of all covariates tested
☐	☒	A description of any assumptions or corrections, such as tests of normality and adjustment for multiple comparisons
☐	☒	A full description of the statistical parameters including central tendency (e.g. means) or other basic estimates (e.g. regression coefficient) AND variation (e.g. standard deviation) or associated estimates of uncertainty (e.g. confidence intervals)
☐	☒	For null hypothesis testing, the test statistic (e.g. F, t, r) with confidence intervals, effect sizes, degrees of freedom and P value noted<br>*Give P values as exact values whenever suitable.*
☒	☐	For Bayesian analysis, information on the choice of priors and Markov chain Monte Carlo settings
☒	☐	For hierarchical and complex designs, identification of the appropriate level for tests and full reporting of outcomes
☒	☐	Estimates of effect sizes (e.g. Cohen's *d*, Pearson's *r*), indicating how they were calculated

Our web collection on statistics for biologists contains articles on many of the points above.

Software and code

Policy information about availability of computer code

Data collection	EPU 1.12 software (Thermo Scientific) was used for CryoEM data collection. Amber18 (University of California, San Francisco) was used for molecular dynamics simulations.
Data analysis	Data have been analysed using the following software: MotionCorr2, Relion 2.0, Scipion2.0, Eman2, RANSAC, PyMOL 2.2.0, Chimera 1.14, iMODFIT 1.51, I-TASSER 5.1, PSIPRED 4.0, KORP, PyRosetta 3.0, PHENIX 1-19-4092-000, REFMAC 5.8.0258 and Malvern MicroCal PEAQ-DSC 1.61. BLAST 5.0 and Espript 3.0 for protein sequence alignments. GraphPad Prism 8.3.0 for preparation of figures and statistics. Trajectories from molecular dynamics simulations were analyzed with cpptraj as part of AmberTools20.

For manuscripts utilizing custom algorithms or software that are central to the research but not yet described in published literature, software must be made available to editors and reviewers. We strongly encourage code deposition in a community repository (e.g. GitHub). See the Nature Research guidelines for submitting code & software for further information.

Data

Policy information about availability of data

All manuscripts must include a data availability statement. This statement should provide the following information, where applicable:

- Accession codes, unique identifiers, or web links for publicly available datasets
- A list of figures that have associated raw data
- A description of any restrictions on data availability

Cryo-EM data have been deposited in the Electron Microscopy Data Bank under accession codes EMD-11624 for full length apo-TH, EMD-11467 [https://www.ebi.ac.uk/emdb/EMD-11467] for full length TH(DA), EMD-11587 [https://www.ebi.ac.uk/emdb/EMD-11587] for CD+OD domains of apo-TH, EMD-11309 for CD+OD domains of TH(DA) and EMD-11467 [https://www.ebi.ac.uk/emdb/EMD-11467] for THNΔ35. The associated atomic models have been deposited in the Protein Data Bank under accession codes 7A2G [https://www.rcsb.org/structure/unreleased/7A2G] for full length apo-TH, [https://www.rcsb.org/structure/6ZVP]

for full length TH(DA), 6ZZU [https://www.rcsb.org/structure/6ZZU] for apo-TH, CD+OD domains, 6ZN2 [https://www.rcsb.org/structure/unreleased/6ZN2] for TH (DA), CD+OD domains and [https://www.rcsb.org/structure/unreleased/7PIM] for THN△35. The Web-link for other structures used in this work are 1TOH [https://www.rcsb.org/structure/1TOH], 2XSN [https://www.rcsb.org/structure/2XSN], 2MDA [https://www.rcsb.org/structure/2MDA], 5PAH (https://www.rcsb.org/structure/5PAH), 1KW0 [https://www.rcsb.org/structure/1KW0], 6HYC [https://www.rcsb.org/structure/6HYC]. The mass spectrometry proteomics data have been deposited to the ProteomeXchange Consortium [http://www.proteomexchange.org/] via the PRIDE partner repository with the dataset identifier PXD024519. Source data underlying Fig. 5a-f, Supplementary Figures 9a,b and Supplementary Tables 8 and 9 are provided with this paper as a Source Data file. The rest of the data will be made available upon reasonable request to the correspondence authors.

Field-specific reporting

Please select the one below that is the best fit for your research. If you are not sure, read the appropriate sections before making your selection.

[x] Life sciences   [ ] Behavioural & social sciences   [ ] Ecological, evolutionary & environmental sciences

For a reference copy of the document with all sections, see nature.com/documents/nr-reporting-summary-flat.pdf

Life sciences study design

All studies must disclose on these points even when the disclosure is negative.

Sample size	As stated in the Statistics and Reproducibility statement in the manuscript a sample size n = 3 (protein samples from independent purifications for each TH state) was chosen. This sample size was sufficient to allow for testing of statistical significance in the applied biophysical and activity assays. The selected value is based on pilot studies comparing the parameters for the different tyrosine hydroxylase (TH) forms studied (wild-type and truncated forms), as well as on previously published data using this type of analysis on both TH and phenylalanine hydroxylase, an enzyme in the same protein family as TH (Flydal et al. DOI: https://doi.org/10.1016/j.biochi.2020.12.002; https://doi.org/10.1073/pnas.1902639116).
Data exclusions	No data were excluded from the analysis.
Replication	The biophysical and activity experiments were replicated at least 3 times with different samples in independent experiments, obtaining successful replications
Randomization	Randomization is not common for these type of studies with small number of samples, where randomization is not a guaranty of group equality of background variables. In any case we alter the order in which the different groups are analyzed by varying their relative location in autosamplers and plates, and the protein samples are handled in the same way before measurements to ensure that we do not introduce other variables.
Blinding	In the biophysical and activity assays blinding is not considered relevant since the assays are prepared and carried out at exactly the same conditions for all the protein samples, and the results are numerical read-outs that are recorded and associated to each sample without human intervention and minimal risk of bias.

Reporting for specific materials, systems and methods

We require information from authors about some types of materials, experimental systems and methods used in many studies. Here, indicate whether each material, system or method listed is relevant to your study. If you are not sure if a list item applies to your research, read the appropriate section before selecting a response.

Materials & experimental systems

n/a	Involved in the study	
[x]	[ ]	Antibodies
[x]	[ ]	Eukaryotic cell lines
[x]	[ ]	Palaeontology and archaeology
[x]	[ ]	Animals and other organisms
[x]	[ ]	Human research participants
[x]	[ ]	Clinical data
[x]	[ ]	Dual use research of concern

Methods

n/a	Involved in the study	
[x]	[ ]	ChIP-seq
[x]	[ ]	Flow cytometry
[x]	[ ]	MRI-based neuroimaging
